# Bioinspired and Bioderived Aqueous Electrocatalysis

**DOI:** 10.1021/acs.chemrev.2c00429

**Published:** 2022-11-10

**Authors:** Jesús Barrio, Angus Pedersen, Silvia Favero, Hui Luo, Mengnan Wang, Saurav Ch. Sarma, Jingyu Feng, Linh Tran Thi Ngoc, Simon Kellner, Alain You Li, Ana Belén Jorge Sobrido, Maria-Magdalena Titirici

**Affiliations:** † Department of Materials, Royal School of Mines, Imperial College London, LondonSW7 2AZ, England, U.K.; ‡ Department of Chemical Engineering, Imperial College London, LondonSW7 2AZ, England, U.K.; § School of Engineering and Materials Science, Queen Mary University of London, LondonE1 4NS, England, U.K.; ∥ Advanced Institute for Materials Research (WPI-AIMR), Tohoku University, 2-1-1 Katahira, Aobaku, Sendai, Miyagi980-8577, Japan

## Abstract

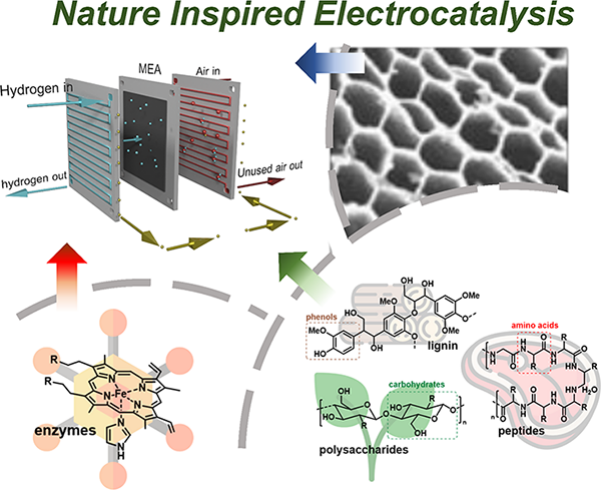

The development of efficient and sustainable electrochemical systems able to provide clean-energy fuels and chemicals is one of the main current challenges of materials science and engineering. Over the last decades, significant advances have been made in the development of robust electrocatalysts for different reactions, with fundamental insights from both computational and experimental work. Some of the most promising systems in the literature are based on expensive and scarce platinum-group metals; however, natural enzymes show the highest per-site catalytic activities, while their active sites are based exclusively on earth-abundant metals. Additionally, natural biomass provides a valuable feedstock for producing advanced carbonaceous materials with porous hierarchical structures. Utilizing resources and design inspiration from nature can help create more sustainable and cost-effective strategies for manufacturing cost-effective, sustainable, and robust electrochemical materials and devices. This review spans from materials to device engineering; we initially discuss the design of carbon-based materials with bioinspired features (such as enzyme active sites), the utilization of biomass resources to construct tailored carbon materials, and their activity in aqueous electrocatalysis for water splitting, oxygen reduction, and CO_2_ reduction. We then delve in the applicability of bioinspired features in electrochemical devices, such as the engineering of bioinspired mass transport and electrode interfaces. Finally, we address remaining challenges, such as the stability of bioinspired active sites or the activity of metal-free carbon materials, and discuss new potential research directions that can open the gates to the implementation of bioinspired sustainable materials in electrochemical devices.

## 1 Introduction

**Figure 1:**
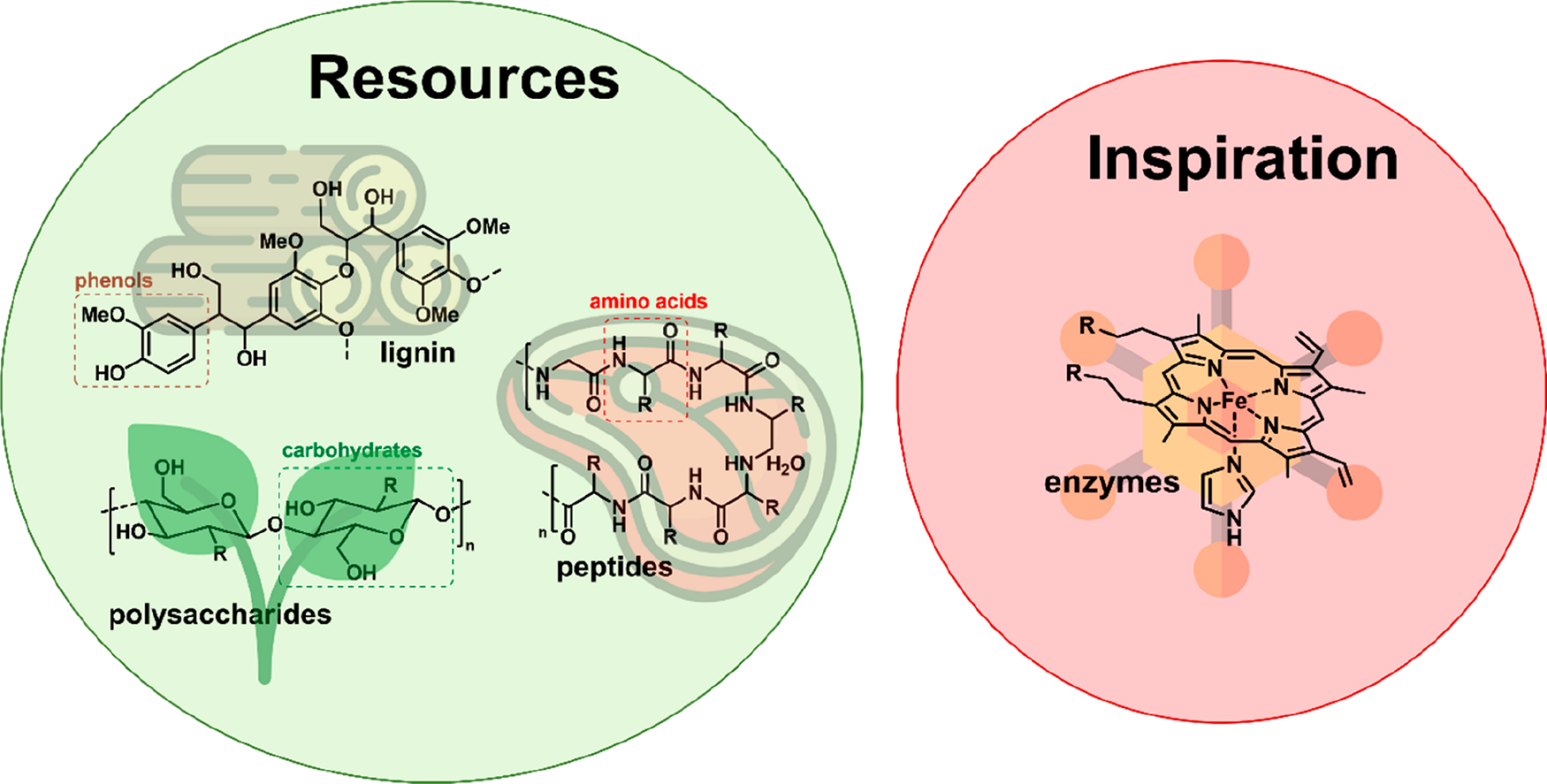
Schematic representation of the different sources of biomass and bioinspiration present in nature.

Our CO_2_ emissions are on a constant rise, reaching a monthly average of 419 ppm in 2021, a record high in the last 2 million years.^1^ In response, many countries have committed to net-zero emissions by 2050 (The EU Green Deal^2^ and the UK 2019 pledge to net zero^3^) or by the latest 2060 (i.e., China).^4^ Consequently, decarbonizing the global economy via the implementation of sustainable and environmentally benign technologies across all sectors has become a main priority for the benefit of future generations. The energy sector contributes to around three-quarters of global greenhouse gas emissions today and will play a pivotal role in averting climate change.^5^ Decarbonizing the energy sector calls for a complete transformation of energy production, transport, and consumption, where shifting away from fossil fuels is key. The development of renewable energy plants, such as solar power, wind power , and hydropower, could deliver a sustainable and carbon neutral electricity system, bring opportunities for decarbonization by electrification.^6^ However, although sectors such as light-duty transportation can potentially be entirely electrified, in other hard-to-abate sectors, such as steel and chemical production, the electrification shares are predicted to remain below 70% by 2050.^7^ For example, processes such as the anthraquinone process for H_2_O_2_,^8^ the Haber–Bosch process for NH_3_,^9^ or methane steam reforming for H_2_^10^ rely on heavily centralized carbon-intensive infrastructures. The Haber–Bosch process alone accounts for 1.3% of the global CO_2_ emissions, contributed to 2% of the world’s total energy consumption,^11^ and requires transportation to the consumption point. An electrochemical approach, however, could help solve these issues by allowing on-site production such as either electrified power-to-X (X = fuels, chemicals) or on-site electricity generation through energy carriers (e.g., H_2_). Commonly explored electrocatalysis for decarbonized energy conversion technologies include green H_2_ production via water electrolysis,^12,13^ power generation from fuel cells,^14^ chemical manufacturing through CO_2_ reduction,^15,16^ H_2_O_2_ production from the oxygen reduction reaction (ORR),^17^ and NH_3_ synthesis by N_2_ reduction.^18,19^ All of these technologies require efficient electrocatalysts to decrease the activation energy barrier and efficiently drive the reactions. Although research in these fields has made significant progress in terms of improving their energy efficiency,^20−22^ so far, many of these electrocatalysts (particularly those involved in water splitting, the hydrogen oxidation reaction (HOR), and ORR) require critical precious metals. For instance, Pt/C is the benchmark catalyst for both ORR in a fuel cell^23^ and the hydrogen evolution reaction (HER),^24^ and Ir-based catalysts are currently irreplaceable for the oxygen evolution reaction (OER) in proton-exchange membrane (PEM) water electrolyzers.^25,26^ These precious metals have been included in the EU’s latest report on critical raw materials,^27^ meaning their natural reserves are depleting and will not sustain the demand in the long term.^28,29^ These uncertainties have largely increased the risk in the catalyst material supply chain, inducing volatility in the commodity prices of Pt and Ir that can significantly impede the large-scale PEM electrolyzer deployment rate. In an analysis performed by Jaramillo and co-workers, they pointed out that current Pt production will limit the PEM electrolyzer capacity to 100 GW/year, and that of Ir will be limited to 2 GW/year, far from the Terawatt (TW) target we need to reach by 2050.^29^ It is also very energy-intensive to source and manufacture these raw materials; 10% of the total global energy-related greenhouse gas (GHG) emissions in 2018 came from the primary production of minerals and metals, with Pt standing out as one of the most GHG-intense metals (more than 10 tons of CO_2_ emitted per kg).^30^ To secure a sustainable future, one has to look for synergy with nature to find environmentally friendly solutions. Nature can potentially provide solutions to energy conversion technologies (including water splitting, ORR, and CO_2_ reduction) in different ways, such as providing both a natural feedstock (biomass) that can be employed for the preparation of advanced carbonaceous catalysts^31^ and models of highly efficient electrochemical systems (enzymes) (Figure 1).

### 1.1 Bioinspired and Bioderived Catalysts

Biomass is a renewable and abundant natural resource that includes agricultural and forestry residues and municipal food waste. Biomass has been recognized as an ideal renewable resource substitute to fossil fuels,^32^ with an estimated worldwide production of approximately 100 billion metric tons per year.^33^ Exploiting biomass can help address concerns related to the availability of raw materials for advanced materials manufacturing and reduce CO_2_ emissions resulting from the mining and manufacturing of materials.^30,34^ For instance, we performed a life cycle assessment that compared the hard carbon anode for a Na-ion battery synthesized from a biomass precursor to commercial graphite (used in Li-ion batteries).^35^ The results show that the former displays significant savings up to 31% in terms of the potential global warming impact.^35^ It can also create important economic revenues due to the wide availability of biomass worldwide, helping farmers and bridging agriculture, waste, and forestry with the materials and chemical industries.

**Figure 2:**
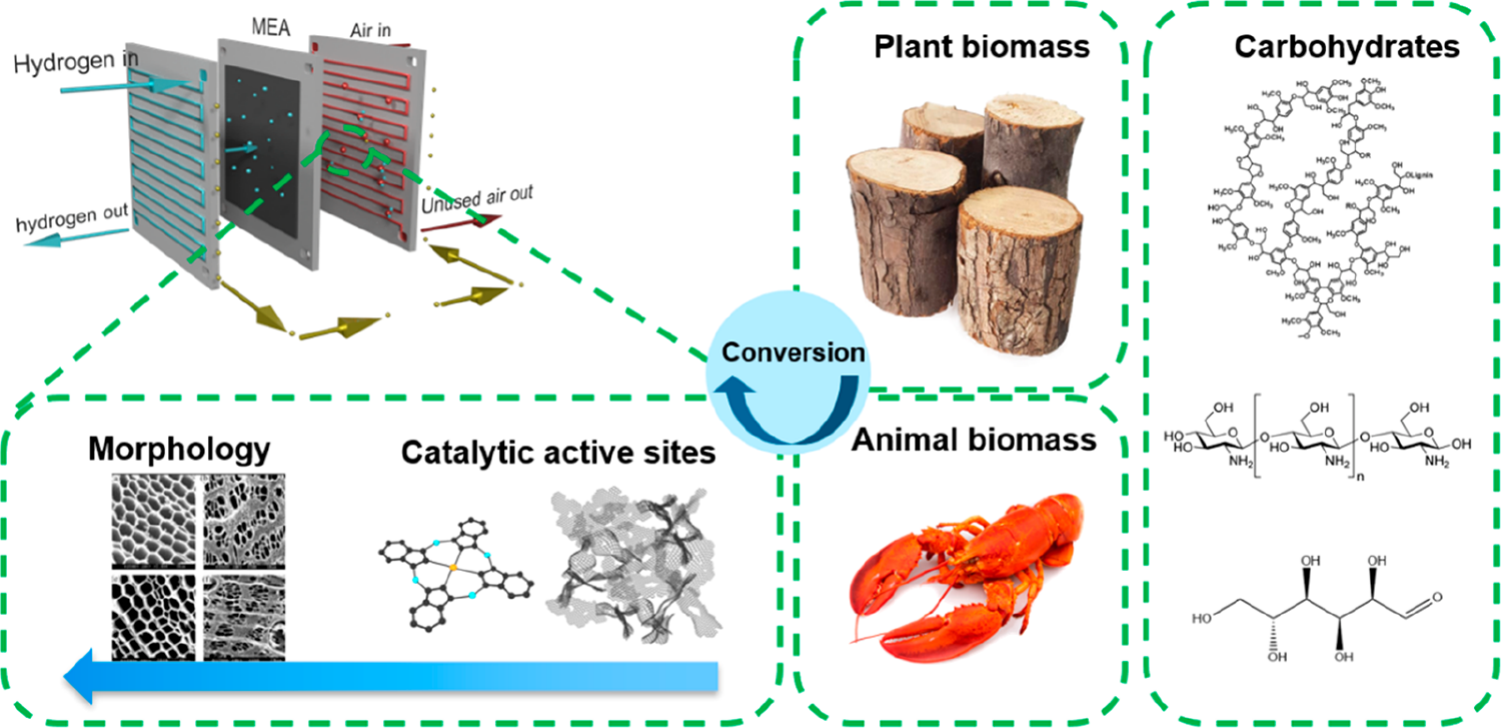
Schematic of biomass types and the active sites in the cathode side of the fuel cell. Figures reproduced with permission from ref (86). Copyright 2010 Hosowaka Powder Technology Foundation.

The production of advanced materials and chemicals from raw biomass has been widely investigated, and several relevant review articles have been published on the topic.^36−40^ The complexity of raw biomass can be exploited to prepare carbonaceous materials with aligned channels, fractal structures, and tunable properties, as well as polymers and other nanomaterials.^41−44^ Two types of biomass precursors have been employed for the synthesis of carbon-based materials: plant biomass (such as lignin from wood and carbohydrates) and animal biomass (such as chitin from shrimps, Figures 1 and 2).^45^ Carbohydrates are comprised of monosaccharides (C_6_ such as glucose, fructose, and galactose or C_5_ such as xylose, arabinose, etc.), disaccharides (maltose, sucrose, lactose, etc.), and polysaccharides (starch, chitin, chitosan, cellulose, etc.), and can be used as carbon-support precursors. Using hydrothermal carbonization or direct pyrolysis, they can be transformed into different types of carbons (amorphous or graphitized)^46^ through complex cascades of dehydration and condensation reactions.^36^ Both the precursor and the heat treatment conditions will influence the chemical composition, surface chemistry, surface area, and pore structure of the resulting carbon.^47^ Additionally, the diversity of raw biomass makes it very compositionally variable depending on where it is extracted from, affecting the composition and reproducibility of the final material. For instance, the chemical composition of lignin and cellulose varies greatly depending on their sources (hard wood or soft wood) and the extraction method.^48,49^ Plant biomass is easier to use when separated in its constituent components, namely, cellulose, hemicellulose, and lignin, by employing biomass fractionation techniques such as the lignoboost process or different organosolv and ionosolv processes.^50−54^

Nevertheless, biomass precursors with natural hierarchical structures can be exploited to optimize electrolyte transport to active sites,^55^ maximizing current density. For instance, employing a wood or bone precursor with a natural hierarchical structure leads to well-defined morphologies and also provides nitrogen moieties derived from the organic collagens.^56−63^ These nitrogen-containing biomass-derived species can form complexes with transition metals and generate carbon-embedded MN_*x*_ (M = metal) catalytic sites resembling those of heme (Figure 1) and part of the enzymatic active site in cytochrome c oxidase (CcO).^64−66^

Nature’s enzymes, such as CcO, have evolved over millions of years into highly efficient mechanisms and pathways that convert abundant atmospheric molecules such as CO_2_ and N_2_ using abundant metal active sites (Fe, Mn, Ni, and Cu) to produce essential chemicals for life, such hydrocarbons and ammonia.^67,68^ Enzymes display remarkable selectivity and turnover numbers toward certain chemical reactions, and no heterogeneous electrochemical catalyst is currently able to compete with enzyme activity and selectivity.^69−71^ Their remarkable efficiency lies primarily in their well-defined active sites and finely tuned surrounding structure, which allows high activity and selectivity by controlling the reactivity of the active site. Additionally, their outer coordination sphere often consists of a peptide matrix with defined channels that allows the efficient and selective transport of reactants (such as H^+^ and electrons) to the active site.^72^ Unsurprisingly, both the active sites and secondary structures of enzymes have been of inspiration for the development of electrocatalysts with a rationally designed interface and enhanced catalytic performance.^73−76^ However, enzymes cannot tolerate harsh pressure or temperature conditions or highly acidic or alkaline pH levels.^77^ Emulating similar structures on more robust materials would benefit the design of new electrocatalysts, improving their selectivity for electrochemical reactions. Biomass-derived materials could potentially resemble the active sites of enzymatic systems while exhibiting conductivity. For example, hemoglobin, which is derived from animal biomass, was recently employed to construct ORR catalysts, as it contains FeN_5_ sites, and has been used as either a catalyst after pyrolysis,^78^ or a doping agent^79^ to hybridize FeN_*x*_ sites into other conductive carbon frameworks. In terms of the transport of reactants, nature also displays unique hierarchical structures that combine different pore sizes across the length scale, which is highly desirable to enable a higher active-site density.^80^ The branching of plants, the vascular networks of animals, the lungs of mammals, and the spiracles of insects are some examples of hierarchal structures.^81,82^ Imitating such structures for the fabrication of electrocatalysts for different reactions has consequently garnered plenty of attention within the last few years.^83−85^

As highlighted above, nature provides a wide set of tools for fabricating the next generation of catalysts for use in energy-conversion technologies (Figure 2). Biomass is a precursor for advanced carbonaceous materials, enzymes are an inspiration for active sites, and hierarchical structures are an inspiration for efficient mass transport. In this review, we discuss in-depth the benefits of nature-inspired aqueous electrocatalysis, focusing on water splitting, ORR, and CO_2_ reduction reactions. We cover the state of the art of biomass-derived carbon materials and bioinspired electrochemical systems and how can we emulate nature employing its own resources. The article will be divided into different electrocatalytic processes: water splitting (HER and OER), the hydrogen oxidation reaction (HOR), the ORR, and CO_2_ reduction. We then delve into the solid–liquid interface control in electrochemical systems resembling the secondary structures of enzymes, and we summarize how nature has aided the development on proton-exchange membranes and flow fields, which are essential components of electrochemical cells. Finally, we provide perspectives for the future of nature-inspired electrocatalysis.

## 2 Biomass-Derived and Bioinspired Water Splitting

Green hydrogen production, defined as hydrogen produced via electrochemical water splitting, is one of the most important energy vectors in our transition to net-zero emissions. There are three major technologies for low-temperature water electrolysis: (1) industrial alkaline water electrolyzers, which use Ni-based alloy electrodes separated by a diaphragm working in highly basic media; (2) PEM water electrolyzers (also known as polymer–electrolyte membrane water electrolyzers) with a Pt-based cathode and an Ir/Ru-based anode separated by a perfluorated sulfonic membrane; and (3) anion exchange membrane (AEM) water electrolyzers, which employ a hydroxide-conducting membrane sandwiched by two Ni-based electrodes.^87,88^

**Figure 3:**
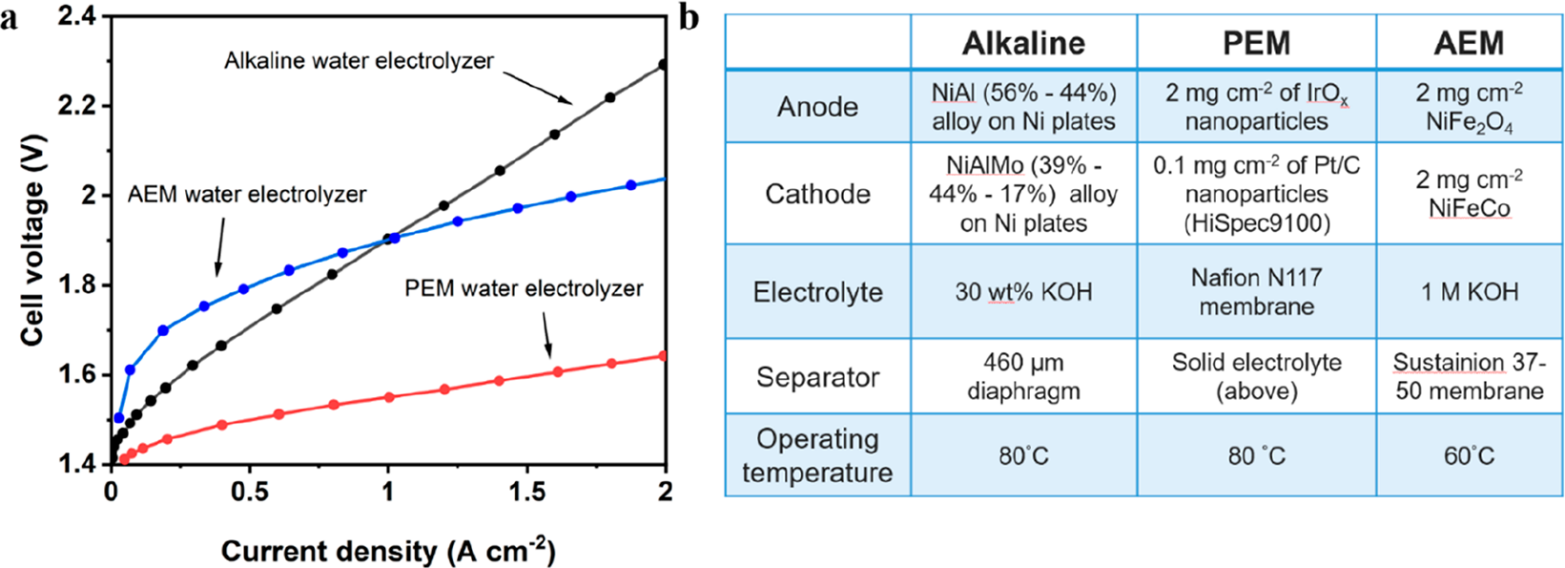
(a) Comparison of typical voltage–current characteristics of the alkaline (black), PEM (red), and AEM (blue) water electrolyzers in a two-electrode configuration. (b) Summary of the cell components and testing conditions. Adapted from refs (93) and (94).

Chemical reactions taking place in acid media are written below, where * represents an available active site for the reactant or intermediate to be adsorbed.^89^Anode:
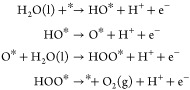
Cathode:
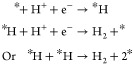


Under alkaline conditions, the chemical reactions are written as shown below.^90^Anode:
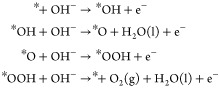
Cathode:
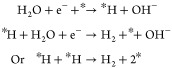


Although a conventional alkaline system can utilize transition-metal catalysts, its current densities still fall below those of a PEM system due to the considerable Ohmic loss caused by the large gap between the two electrodes and the thickness of the diaphragm. However, only Ru- or Ir-based catalysts can sustain the highly acidic working environment of PEM water electrolyzers, as carbon-based supports are not suitable due to carbon oxidation,^91^ making it difficult to widely deploy this technology. The recently developed AEM water electrolyzers that use nonprecious metals have exhibited comparable performance,^92^ demonstrating the great potential for upscaling. Nonetheless, further research is required to develop sufficient membranes and enhance the durability of such systems.^92^Figure 3 shows polarization curves of these three cells, where the PEM system exhibits the highest efficiency.

In PEM water splitting systems, while HER can occur at minimum overpotentials with low Pt loadings, OER requires much higher overpotentials. With a Pt loading of 0.05 mg cm^–2^ in PEM electrolyzer, it is possible to achieve an HER overpotential below 2 mV,^95^ but more than 0.3 mg cm^–2^ IrO_*x*_ is required to sustain an overpotential below 440 mV on the OER side.^96^ The overall performance is mainly suppressed by the sluggish anodic OER as the limiting factor. OER is thus one of the most researched reactions in decades due to its critical roles in electrocatalytic energy storage and fuel production. Several models have been developed for OER mechanistic descriptors, representatives of which are scaling relations (Sabatier principle) and the lattice oxygen mechanism (associated with structural stability).^89,97^ The Sabatier principle focuses on the free energy of adsorption of an intermediate at the catalyst’s metal surface, while lattice oxygen evolution suggests the participation of lattice oxygen as another important descriptor besides metal-site adsorption on a dynamic surface.^98^ The lattice oxygen mechanism features direct O–O coupling achieved by the evolution of lattice oxygen when the oxide catalyst is destabilized, hence having the potential to bypass the theoretical overpotential set by the scaling relation and being closely linked to thermodynamic instability.^98−101^

### 2.1 Bioderived Catalysts for OER

Metal-doped carbon-based materials have primarily been investigated as hydrogen evolution catalysts, with only a few reports focusing on oxygen evolution in alkaline conditions.^102−104^ Due to their chemical diversity and the inherent presence of heteroatoms, biomass precursors can be utilized to synthesize heteroatom-doped (metallic or nonmetallic) carbons,^105−107^ which is one of the most widespread approaches to introduce active sites via charge redistribution.^108−115^ Their OER performances have been summarized in detail in other reviews.^116,117^ A uniform distribution of transition-metal atoms (Fe, Co, and Ni) in the carbon matrix enhances interfacial charge transfer while also promoting hydroxide accessibility and electronic conductivity, leading to enhanced activity.^107,115,118−121^ This is reflected in previous work, where egg-derived carbon microspheres exhibited low onset potentials (∼1.5 V_RHE_, RHE = reversible hydrogen electrode), a high current density (74.6 mAcm^–2^ at −1.6 V_RHE_), and excellent stability for 20 h with 95% current retention due to a large specific surface area, a high pore volume, and the innate presence of nitrogen, phosphorus, and iron.^107^

It has also been observed that a small percentage of doped N in the carbon matrix can considerably reduce the OER overpotential due to the decreased kinetic barriers and the assisted binding of *OH, *O, and *OOH intermediates.^122^ The introduction of electron donors (P and S), electron acceptors (B), or oxygen defects with different degree of oxidation can also be used to engineer the valence band orbitals of a carbon matrix or facilitate electrolyte infiltration and oxygen desorption.^123−125^

At this stage, it is also important to mention that carbon-based feed stocks (graphite, carbon nanotube, bioderived carbon, etc.), chemical precursors, and common electrolytes (NaOH, KOH, and HClO_4_) might contain metallic impurities, conceivably causing artifacts or completely misleading conclusions for metal-free materials.^126,127^ Purification should be conducted consistently and throughout the whole process from synthesis to control experiments to eliminate the effect of trace metals. Carbon-based materials are also prone to oxidation due to the high oxidation potential applied during catalyst testing. Furthermore, the bubbles produced during the OER process might cause the carbon morphology to collapse. Therefore, the application of carbon-based materials as catalysts for OER remains in its infancy, and the role of carbon-based materials is reduced to acting as hosts for their metallic counterparts.^128^ Consequently, we believe that a fair comparison of their performance with the state-of-the-art catalysts is inaccurate and challenging to achieve. For a more focused comparison of OER performances among different catalysts, we would like to refer the reader to previously published reviews.^116,117^

### 2.2 Bioinspired Catalysts for OER

**Figure 4:**
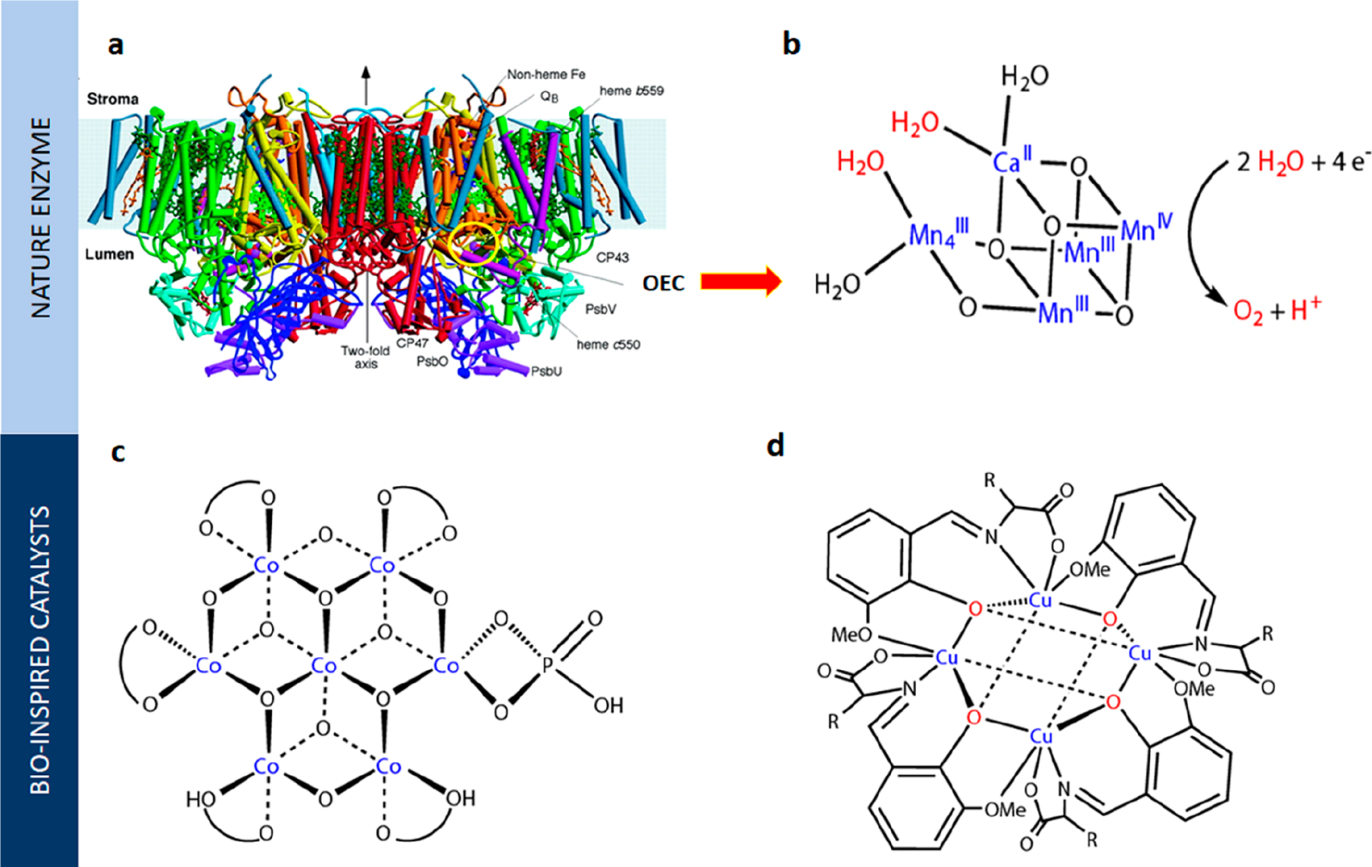
(a) Overall structure of photosystem II. Reprinted with permission from ref (151). Copyright 2004 American Association for the Advancement of Science. (b) Molecular structure of the Mn_4_CaO_5_ OEC. Adapted with permission from ref (136). Copyright Springer Nature 2011. (c) 45°-Rotated view of Co-OEC. Adapted with permission from ref (142). Copyright 2021 American Chemical Society. (d) Cu_4_O_4_ cubane. Adapted with permission from ref (145). Copyright 2018 Wiley-VCH.

In nature, oxygen is produced in plants and algae via the photosynthetic process, which employs the enzyme photosystem II (PSII). This enzyme is constructed by a large homodimer protein complex comprised of many polypeptide subunits and cofactors (Figure 4a).^129^ Upon receiving photons, an electron–hole pair is generated within the enzyme that oxidizes a chlorophyll molecule (P680 → P680^+^) and reduces a pheophytin acceptor. The oxidized molecule subsequently activates the Mn-based oxygen-evolving complex (OEC),^129^ which is the active site for water oxidation (Figure 4b), before returning to its most reduced state.^129,130^ The produced oxygen species are highly toxic to organisms and can cause the removal of the protein within the photosystem and the disassembly of the Mn-based cluster. Upon the incorporation of the newly synthesized protein into the membrane-bound complex,^131^ the OEC is also reassembled. This continual self-healing catalytic process allows precise control of active sites for water oxidation, yields high selectivity, and resembles the dynamic cycle associated with dissolution–redeposition process in the lattice oxygen mechanism.^132^ High-resolution crystallographic analysis of the photosystem revealed the structure of the cubane-like Mn–Mn_3_CaO_5_ cluster (the OEC),^130,133−136^ and density function theory (DFT) models described its reconstruction.^136−138^

As the molecular structure was elucidated, initial efforts were made to synthesize an artificial photosynthesis II system or OECs, but the outcomes have shown limited success.^139−141^ Nevertheless, such inspiration from nature resulted into the discovery of many transition μ-oxo-bridged metal oxide clusters and complexes. In the pioneer work on an artificial leaf,^142^ Nocera prepared a cobalt–phosphate cluster through electrodeposition with edge-sharing octahedral CoO_6_, a structural analog to the OEC in the photosystem II that can split water at neutral and near-neutral conditions. Other cubane-like complexes with similar molecular motifs have been explored for water oxidation, including mixed-metal manganese oxide,^143^ layered organic cobalt phosphonate,^144^ copper oxide,^145^ and octanuclear Cu(II) clusters (Figure 4 c and d).^146^ Since oxygen evolution in nature involves not only the core catalyst but also assistance from the protein backbone, supporting mediators can be added as part of the biomimetic strategy. Li et al. synthesized a carboxylate-incorporated Ni–Fe coordination polymer in which the negatively charged carboxylate ligands not only stabilized the high valence states of metal centers but also served as proton-transfer relays, efficiently reducing the redox potential.^147^ Similarly, introducing an electron-transfer mediator is another viable approach for controlling oxidation-reaction kinetics.^148^ Electron-transport assemblies imitating the charge-transfer function of the tyrosine–histidine pair were found to suppress undesirable recombination and consequently increase the quantum efficiency.^149,150^

### 2.3 Bioderived Catalysts for HER

HER involves a series of elementary steps that take place at the electrode–electrolyte interface. Depending on the pH of the electrolyte, H_2_ is generated via the reduction of either a proton (H^+^ in acidic media) or H_2_O (in alkaline media), as shown in the equations at the beginning of section 2. Since the HER kinetics strongly correlate with the hydrogen adsorption energy (Δ*G*_H_^°^), this factor constitutes a good descriptor of materials that can catalyze HER. Therefore, in 2004, Nørskov’s group calculated the corresponding Δ*G*_H_^°^ on various metals using DFT, which features the volcano plot, and the results perfectly explained the superior HER activity of Pt.^152^ In alkaline media, the kinetics of HER on most metal catalysts is more sluggish compared to that in acidic electrolytes due to a distinct pathway. Due to higher pH, H_2_O must first be dissociated into H^+^, which requires additional energy to drive the overall reaction.^153^ So far, a Pt-based catalyst is still the state-of-the-art HER catalyst in both acidic and alkaline conditions.^95,154^ Nonprecious-metal-based HER catalyst research has also made significant achievements, with several metal sulfides, phosphides,^155,156^ and selenides such as MoS_2_,^157^ CoP,^158^ and WSe_2_ active in acidic conditions and Ni-based catalysts functional in alkaline conditions.^159,160^

Like OER, bioderived materials can be used to facilitate electron transfer and proton diffusion for HER. Since the best-performing metal-based HER catalysts were extensively researched previously, engineering bioderived materials as the carbon support to these active sites constitutes a more suitable strategy. In a previous review, Zhao et al. listed the state-of-the-art HER catalysts in acid and alkaline conditions, with a detailed description of their morphologies and electrochemical performance.^153^ On one hand, the excellent conductivity of the carbon matrix can lead to a higher proton or electron transport rate. On the other hand, templated mesoporous carbon can provide sufficient electrolyte transport channels and promote mass transport, the bubble release of carbon sites, and H* adsorption, improving HER activities.^161,162^ Catalysts formed via in situ templating, such as the 3D coral-like carbon support for Ni_3_S_2_ and the cube-on-sheet matrix for Co(OH)_2_, possess intrinsic hierarchical structures^163,164^ that not only promote gas diffusion but also provide more accessible active sites. In addition, heteroatom-doped carbon can help stabilize metal catalytic sites against leaching or aggregation by coordinating interactions between the heteroatoms and the metals.^165^ Wang et al. made a N–P-doped hierarchically porous carbon matrix from phytic acid and chitosan that can stabilize FeCoP_2_ sites, protecting the active metal sites from acidic corrosion and thus exposing abundant catalytic sites.

### 2.4 Bioinspired Catalysts for HER

Besides the state-of-the art Pt- and several nonprecious-metal-based alterative catalysts described above, nature has also provided inspiration for the design of highly active HER catalysts. Interestingly, proton reduction is a typical reaction of bioenergetic metabolism in many living organisms.^166^ The metalloenzymes responsible for catalyzing such a reaction are called hydrogenases, which work with remarkably high catalytic rates close to the thermodynamic reaction equilibrium.^167^ The nature of the active sites in such hydrogenases and their ability perform a catalytic HER function could be judiciously transposed to artificial nonprecious-metal-centered catalysts to rival Pt. Since these enzymes can also catalyze the reverse reaction, namely, proton oxidation, the inspiration from these enzyme materials can also apply to the HOR, as shown in the following section.

**Figure 5:**
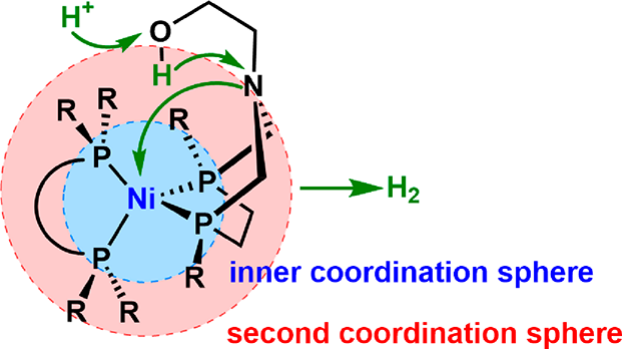
Coordination sphere illustration of a Ni-based molecular catalyst and catalytic reaction diagram for HER. Adapted with permission from ref (168). Copyright 2014 American Chemical Society.

Hydrogenase-inspired molecular electrocatalysts use only relatively inexpensive and highly abundant transition metals, such as Fe, Ni, Mn, and Mo, coordinated to different donor ligands containing basic N or S atoms.^169^ Various coordination spheres can be defined on the catalysts (Figure 5). With Ni-based catalysts, the metal center is reduced prior to protonation, forming a Ni hydride complex. The whole ligand contributes to reducing the activation energy, with the phosphines affecting the reducibility of the Ni center and the dangling hydroxyl group on the ligand helping heterolytic H_2_ formation by promoting intramolecular proton transfer.^168^ This metal–ligand synergy greatly helps reduce the overpotential of the process.^166,170−172^ When these molecular catalysts are loaded onto electrodes, typically carbon nanotubes, they can serve as active HER catalysts in the half-cell reaction.^173^ Pioneering work from the DuBois group has shown the potential of a Ni-based molecular catalyst mimicking hydrogenase for HER, with a high turnover frequency (TOF) of 500 s^–1^.^148,174,175^

Similar to OER, the proton-transfer relay process that happens in the portion backbone in natural hydrogenases can also be imitated by engineering the supporting mediator of the molecular catalysts. Dubois et al. introduced an amine-containing diphosphine ligand for Ni. They showed that the incorporated nitrogen assisted proton transport and resulted in a significant decrease in the activation barrier for dihydrogen bond formation.^148^

**Figure 6:**
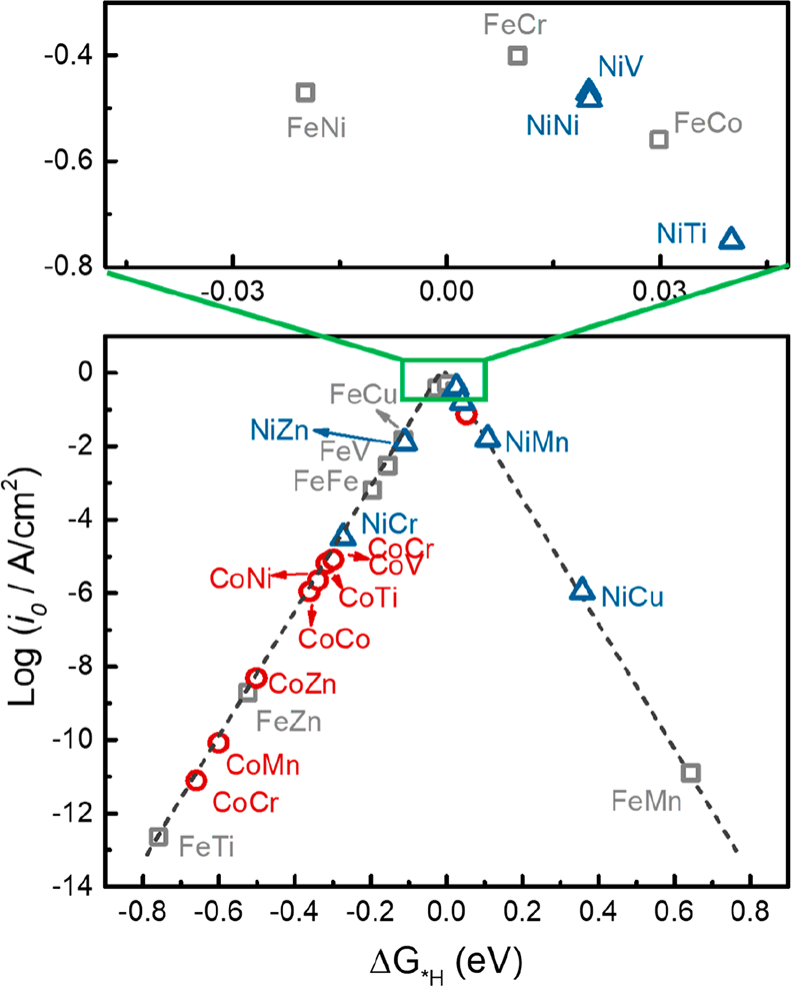
HER volcano curve of the exchange current density (*i*_0_) as a function of MM′-NPG (in-plane dual-metal atom in nitrogen-doped porous graphene). Reproduced with permission from ref (176). Copyright 2021 Elsevier.

Although the above-mentioned molecular catalysts have shown good activity, their low resistance to oxidative or other harsh conditions results in low stability. Catalyst synthesis and electrode fabrication also remain large challenges.^166^ Besides, the large coordination sphere structure (Figure 5) required to drive the catalytic reaction and proton or electron transport has also restricted the active site density per geometric electrode, resulting in low performance in real devices. For instance, Artero and co-workers prepared the same gas diffusion electrode (GDE) with a Ni-centered molecular catalyst and Pt/C for water electrolysis. The former loading can be achieved at 2.5 × 10^–8^ mol_Ni_ cm^–2^, an order of magnitude lower than that of Pt/C (2.5 × 10^–7^ mol_Pt_ cm^–2^). As a consequence, the current density for the Ni catalyst at an overpotential of 100 mV at 25 °C is only 7.1 mA cm^–2^ compared to that of 18.4 mA cm^–2^ for Pt/C.^175^ A strategy to overcome these issues is to design supported single or dual catalysts, where the metal center coordinated by base atoms (i.e., N, S, and P) replicates the active sites in the enzymatic catalysts and can optimize the binding strength of *H and the supporting materials are engineered to enable fast proton and electron transfer. For the synthesis strategy and recent advances, readers may refer to previous reported reviews.^177−184^ By developing a universal design principle to evaluate the activity of graphene-based single-atom catalysts, Xu et al. indicated that the catalytic activity of single-atom catalysts is highly correlated with the local environment of the metal center. The electronic structures of these metal centers are controlled by the coordination number and the nearest-neighbor atoms, affecting the HER performance through the *H binding energy.^185^ Dual-atom catalysts can further modulate the interaction with *H through the synergetic effect between the two metal centers, leading to superior HER activities (Figure 6).^176,183^

**Figure 7:**
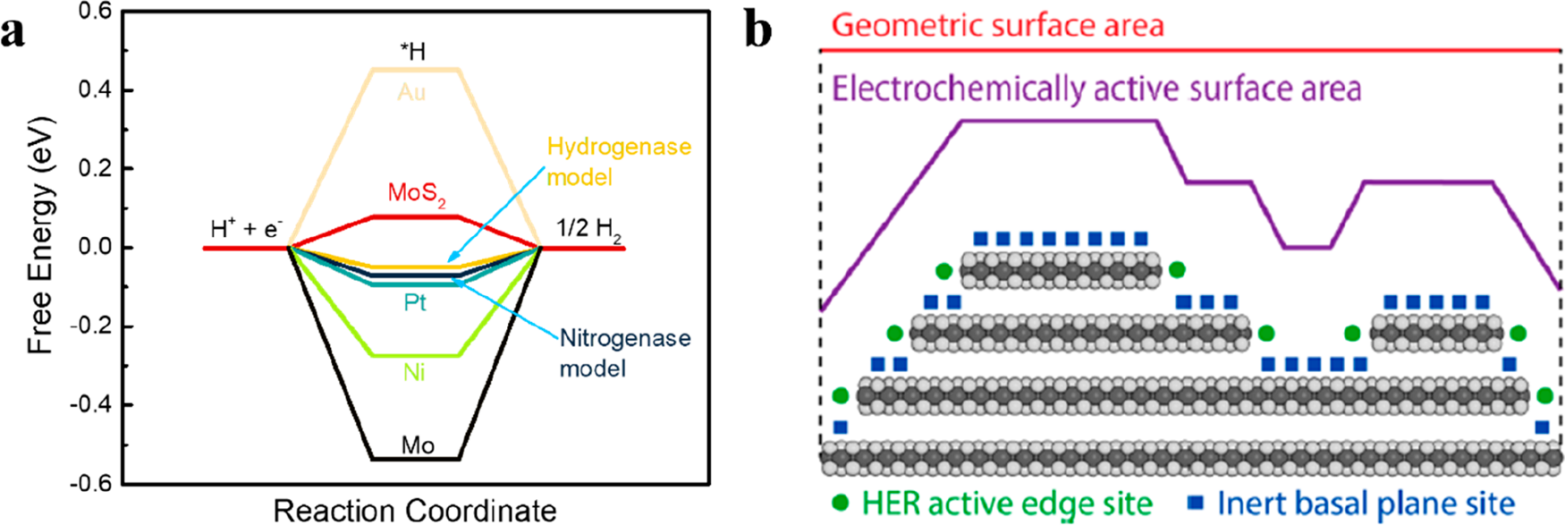
(a) Calculated free-energy diagram for HER for the hydrogenase model, the nitrogenase model, MoS_2_, and metal-surface (Au, Pt, Ni, and Mo) catalysts at a potential *U* = 0 relative to the standard hydrogen electrode (SHE) at pH 0. Reproduced with permission from ref (186). Copyright 2005 American Chemical Society. (B) Two-dimensional representation of MoS_2_ catalyst electrocatalytic active surface area and projected geometric surface area. Reproduced with permission from ref (157). Copyright 2014 American Chemical Society

Besides these molecular and single-atom catalysts, MoS_2_ has also shown exceptional HER performance. MoS_2_ has a low activation energy requirement due to its resemblance to the FeCo cofactor active site found in nitrogenase (Figure 7a), which has a hydrogen binding energy close to that of Pt.^186^ Jaramillo et al. combined experimental analysis with computational methods to identify the active sites in nanoparticulate MoS_2_. They discovered a linear correlation of the HER performance with the number of edge sites on the MoS_2_ catalyst, which were later proved to be the active sites.^187^ They summarized in a review the general synthesis strategies and performance figures of metrics, based on which further development toward increasing the number of accessible active sites per the geometric electrode area has been pointed out (Figure 7b).^157^

Overall, inspiration from nature via photosynthesis led to the successful design of various transition μ-oxo-bridged metal oxide clusters and complexes for OER. Similarly, molecular catalysts and carbon-supported single- or dual-atom catalysts inspired by the active sites in hydrogenases, as well as the MoS_2_ catalyst structure inspired by nitrogenase, have also lead to breakthroughs in HER research.^147−150^ Electrocatalytic active centers can work in conjunction with charge mediators in their coordination sphere to assist proton an electron transfer, akin to natural enzymes. At a macroscopic level, the catalyst scaffold is important for the effective dispersion of active sites and gas diffusion. This can be achieved through a carbon network or by self-construction. Finally, the combination of metal complexes, charge-transfer relays, and hierarchical structures will bring about unprecedented catalytic performance for which a new class of metal–arbon hybrids has been foreseen to emerge in the future.

## 3 Bioderived and Bioinspired Catalysts for ORR and HOR

**Figure 8:**
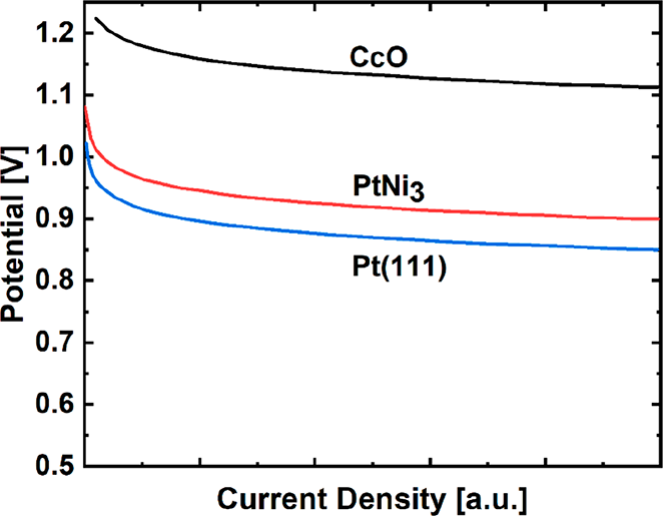
Theoretical polarization curves of the cathode potential versus current density of CcO (black), PtNi_3_ (red), and Pt(111) (blue). Reproduced with permission from ref (195). Copyright 2010 American Chemical Society. Original data of CcO were calculated from ref (196).

Both the switch from gray (steam methane reforming) to green hydrogen (in electrolyzers) and its utilization in fuel cells could bring significant environmental benefits through low carbon emissions and highly efficient energy conversion. In a typical PEM fuel cell, H_2_ gas is supplied to the anode side where it is oxidized into protons, with electrons generated (i.e., HOR) following either Tafel/Volmer or Heyrovsky/Volmer steps as follows:
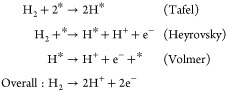


The produced electrons in a PEMFC travel through an external circuit to dispense electrical power, while the protons cross the membrane to the cathode side, where they react with the oxygen stream to produce water (i.e., ORR) in a multistep four-electron-transfer process via the following mechanism:
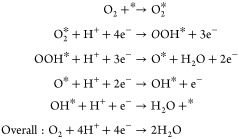


In the case of an alkaline anion-exchange-membrane fuel cell (AEMFC), H_2_ is still oxidized at the anode, although OH^–^ instead of the H^+^ passes through the membrane to react with H_2_. The Tafel/Volmer or Heyrovsky/Volmer mechanisms are as follows:
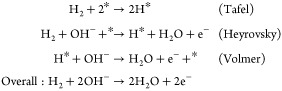


Consequently, at the cathode of an AEMFC, the ORR produces OH^–^:
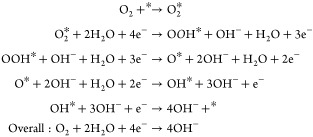


ORR is highly sluggish in its kinetics and can also proceed by a “two-electron” process, generating H_2_O_2_.^188^ An efficient fuel cell ORR electrocatalyst should drive the reaction toward four-electron pathways to provide a high current and high operating potential, since the H_2_O_2_ pathway reduces the achievable current and degrades the membrane.^189,190^ The binding energies in the four-electron ORR process have adsorbed intermediates of the first and third electron or proton transfer step (OOH* and OH*) that are strongly correlated to each other, obeying a linear scaling relationship.^191^ The difference in energy of these intermediates follows a constant of ca. 3.2 eV.^192^ Therefore, at least ca. 1.6 eV (3.2 eV/2) is the minimum energy for each of the two electron or proton transfer steps. As the energy difference between each of the four intermediates ideally amounts to 1.23 eV, a minimum overpotential of ca. 0.3 V can be expected for ORR catalysts with one active site, such as the active sites present in Pt(111) and PtNi_3_ (Figure 8)^193^, the most active ORR catalyst in an aqueous environment to date.^194^ Increasing the number of oxygen binding sites to two via dual-metal atoms in atomic proximity, present in CcO, can enable the optimization of the electronic structures of each binding site such that the binding energy is modified in accordance with the scaling relation, allowing a reduced minimum overpotential (Figure 8).^195^ The possible benefits of bioinspired dual-metal atom sites are further discussed in section 3.3.

In terms of catalysts for fuel cells, Pt nanoparticles supported on carbon (Pt/C) are commonly used commercially, but the high Pt requirement in fuel cells could lead to global supply issues. For instance, assuming an annual production of 1.25 million PEMFC-based vehicles (10 million deployed globally by 2030),^197^ current Pt loadings (0.125 mg_pt,cathode_ cm^–2^ and 0.05 mg_pt,anode_ cm^–2^, 1165 mW cm^–2^, 87.9 kW gross power)^198^ require ∼12.4 tons of Pt annually (assuming 25% Pt recycling), therefore alone consuming ∼7% of the global Pt produced annually (180 tons of Pt produced in 2021).^199^ Combined with other developing fuel cell applications (buses, trains, boats, aviation, back-up power, and electrolyzers), this would exceed the reasonable annual consumption of 10% of an element for a new technology.^200^ Moreover, as mentioned, the high loading of Pt-group catalysts is identified as the one significant barrier to reducing cost, accounting for 40–50% of the cost of a fuel cell (at 500,000 units yr^–1^).^201^ Pt is also prone to cost fluctuations and limited accessibility, further hindering its commercialization in fuel cells. Besides, Pt also suffers from CO poisoning and methanol crossover.^202^ To address these issues, significant progress has been made to lower the Pt loading, including designing non-Pt metal and nonmetal heteroatom-doped carbon catalysts.^64,203^ Strategies such as increasing the density and intrinsic activity of active sites,^204^ constructing hierarchical structures, and enlarging triple-phase boundaries have improved both electrocatalytic activity and stability.^203^ In the sections below, we will discuss how catalysts have so far been inspired or derived from nature along with how nature can continue to direct future research directions toward active and stable electrocatalysts.

### 3.1 Bioderived Metal-Free Catalysts for ORR

**Figure 9:**
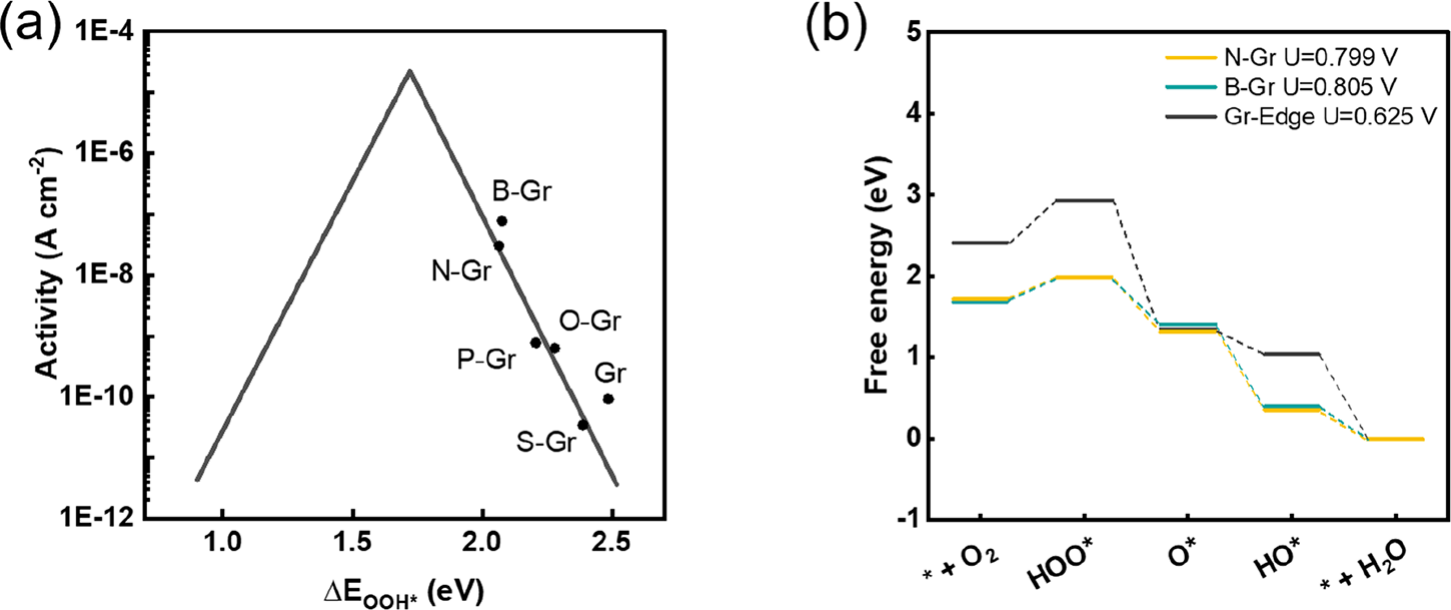
(a) Volcano plot of activity versus OOH* binding free energy (G_OOH*_) for heteroatom-doped graphene. (b) Free-energy diagrams for graphitic N-doped graphene (N-Gr, *U* (limiting potential) = 0.799 V), graphitic boron-doped graphene (B-Gr, *U* = 0.805 V), and carbon on graphene edge (Gr-Edge, *U* = 0.625 V). Panels a and b were adapted from ref (203).

Biomass in electrocatalysis is often used as a precursor for the formation of conductive carbon supports with high surface areas and ORR activities in alkaline conditions.^203^ Bioderived catalysts have been successfully prepared from numerous materials, including wood,^56−59^ sisal leaves,^205^ pine needles,^206^ rice husks,^207^ bamboo,^208^ loofahs,^209^ watermelon,^210^ pomelo peels,^211^ hemp,^212,213^ clover,^214^ peanut skin,^215^ fern fiber,^216^ bones,^60−63,205^ leather,^217^ shrimp shells,^218^ and even butterfly wings.^219^ Clearly some of these sources are not practically suitable due to limited supplies (an example is discussed further in section 3.2) and environment destruction. Biomass-derived carbon sources with heteroatoms have been employed to help with the formation of active sites or to form the active site itself. B- or N-doped carbons have shown better activity as active sites for ORR compared to oxygen, sulfur, and carbon edges, as shown by volcano plots (Figure 9). In addition, B- and N-doped carbons share similar free-energy diagrams, while carbon edge sites require a much larger driving force to complete their four-electron pathway (Figure 9b). Via a simple heat treatment under inert gas, the heteroatoms originating from biomass could hybridize into the carbon framework. Meanwhile, NH_3_ activation and the addition of melamine, urea, or dicyandiamide have become common methods to introduce or further add nitrogen and improve activity.^220^ The type of a particular dopant element introduced will affect the performance,^220^ although the true active sites in metal-free catalysts are often debated, as defects in the carbon framework have experimentally been shown to provide greater catalytic activity than nitrogenated sites.^221^ An understanding of the most active and stable heteroatoms or defects would help form the criteria for the targeted synthesis of active sites used in metal-free carbon-based electrocatalysts.

Despite the many publications on this topic,^222,223^ the performance of heteroatom-doped carbons is still too kinetically hindered in the four-electron ORR pathway to produce electricity in practical fuel cell devices. However, they have demonstrated the selective two-electron pathway to H_2_O_2_ production through heteroatom doping such as nitrogen^224^ and phosphorus.^225,226^ The H_2_O_2_ production market is estimated to be worth 6 billion dollars by 2023,^227^ opening opportunities for the production of metal-free carbon electrocatalysts from biomass. However, the heteroatom amount and species vary depending on the source, significantly reducing the reproducibility of biomass-derived catalysts.^228^ Using purified bioderived precursors to prepare catalysts can help tackle this irreproducibility issue.^229^ However, extracting raw pure precursors from biomass such as carbohydrates and lignin adds an additional synthetic step, thus increasing the price of the catalyst while decreasing its overall sustainability because of the harsh conditions required for biomass fractionation (KOH, sulphites, or organic solvents).^229^ Therefore, if cheap and reproducible biomass-derived catalysts can be obtained, they can be practically implemented in H_2_O_2_ production devices.

### 3.2 Bioderived Non-PGM-Based Catalysts for ORR

Although to date Pt and platinum-group metal (PGM) alloys work best in both acid and alkaline conditions, the design of PGM-free catalysts is a promising method to replace the scarce and expensive PGM materials. So far, the most widespread PGM-free catalysts are transition-metal nanoparticles and single sites loaded on carbon substrates with or without nitrogen (M–C and M–NC; M = Fe, Co, Ni, Mn).^230,231^ While metal clusters aggregate easily during long-term operation, chelating nitrogen atoms around single metallic species leads to sites more resistant to alkaline and acid leaching.^232^ Unfortunately, most biomass sources do not provide a sufficient amount of heteroatoms (nitrogen or others) for proper metal single-site chelation,.^228^ However, chitosan from crustaceans, with its naturally high N content, has shown promise as a suitable nitrogen-doped carbon-biomass-derived precursor to host non-PGM ORR catalysts.^233^ To understand if sufficient chitosan (and other potential biomass sources) could be supplied for electrocatalyst applications, we look at the cathode catalyst in PEMFC light-duty vehicles. We again use our previously calculated assumed global annual production of 1.25 million PEMFC light-duty vehicles,^197^ each with an fuel cell active area of 9.9 m^2^ (as estimated in the Toyota Mirai).^234^ Assuming that state-of-the-art non-PGM cathode catalysts could provide the required fuel cell power and durability at 4 mg_carbon_ cm^–2^ (reasonable considering recent developments^235^), this results in 495 tonnes per annum of non-PGM catalyst required for the PEMFC cathode. Assuming a 20% process yield from precursor materials results in ∼2500 tonnes of catalyst precursors required annually. Chitosan-derived catalysts have not provided state-of-the-art non-PGM catalyst performance, so one can expect to require at least 10× more of the chitosan-derived catalyst to reach an equivalent performance. Chitosan comprises between 10% and 25% dry weight of crustaceans, with 8.4 million tonnes of crustacean shell estimated to be produced as waste in 2017;^236^ this demonstrates the large production capability of chitosan, which could fulfill new applications such as electrocatalysis. However, real market production quantities of chitosan are much lower, with a projected market size of 21,400 tonnes for 2015, and there are no known industrial chitosan production facilities to date for pure chitosan, leading to high costs.^237,238^ This highlights some issues with the use of biomass feedstocks for scalable synthesis methods.

Carbohydrates such as glucose and gelatin are cheap, abundant, and do not require complex synthesis processes and pretreatment, making them suitable precursors for single-atom M–NC catalysts on a large scale; however, they would require an additional dopant, such as N, to be introduced to assist the formation of chelating metal sites. Another issue in biomass-derived materials for ORR lies in the difficulty of achieving defined highly active catalytic sites such as single-metal-atom MN_*x*_ sites.^239,240^ During synthesis, MN_*x*_ sites tend to aggregate into particles to form carbides or oxides with the carbon skeleton during typical pyrolysis, hence reducing the catalytic activity and stability.^241,242^ To avoid this, efforts should be focused on first synthesizing pyrolyzed heteroatom-doped carbon from biomass and then subsequently incorporating metal ions to avoid the undesirable carbothermal reactions at high temperatures.^21,243−246^

Researchers have intensively studied the influence of the structure,^247^ evolution pathways,^248^ and degradation mechanism^249^ and made comparisons between different single-atom M–NC sites,^249,250^ which established a fundamental understanding of the active sites. Interestingly, highly active MN_*x*_ sites can be found in nature within CcO or hemoglobin.^195^ Therefore, in the next section we expand on the design concept of an active site inspired by nature to create next-generation ORR catalysts.

### 3.3 Bioinspired Design of Active Sites in ORR Catalysts

Pt is highly efficient toward HOR in PEMFCs, requiring only ultralow loadings (<10 μg_Pt_ cm^–2^) to proceed effectively.^251,252^ However, at these ultralow loadings the catalyst becomes highly sensitive to fuel contaminants such as CO and H_2_S, which irreversibly poison Pt.^253^ Alternative hydrogenase-inspired Ni-based catalysts, previously discussed for HER in section 2.2 and illustrated in Figure 6, can display tolerance to these contaminants, thereby lowering fuel cell stack and H_2_ purification costs. For instance, a molecular [Ni(P_2_^Cy^N_2_^CH_2_pyrene^)_2_](BF_4_)_2_ complex attached to multiwalled carbon nanotubes (MWCNTs) on a gas diffusion layer electrode maintained a constant H_2_ oxidation current (at 0.25 V vs normal hydrogen electrode (NHE)) in a 50 ppm CO atmosphere over 80 min, while a commercial Pt electrode (0.5 mg_Pt_ cm^–2^) became completely poisoned within 40 min.^254^ In terms of power output, a biomimetic Ni bisdiphosphine complex ([Ni(P_2_^Cy^N_2_^Arg^)_2_]^7+^) immobilized on modified carbon nanotubes at the anode (and Pt at the cathode) reached 14 mW cm^–2^ (at 0.47 V and 60 °C) in a PEMFC, only six times less than a comparably constructed full-Pt-based PEMFC.^255^ Further optimizing the testing of a similar Ni-based complex, Artero and co-workers recently achieved up to 83 ± 6 A mg_Ni_^–1^ in a gas diffusion layer (at 55 °C and 0.4 V overpotential), a mass activity <1 order of magnitude lower than those of state-of-the-art ultralow-loading Pt-based anodes.^256^ This signifies the possible practical application of these complexes, assuming that bubble accumulation can be overcome,^256^ long-term stability can be demonstrated, and a scalable synthesis approach can be developed. Successful attempts at incorporating bioinspired catalysts at the anode in AEMFCs have not, to the best of our knowledge, been reported to date, likely due to the slow HOR kinetics in alkaline conditions. The difficultly of HOR in AEMFC was demonstrated Davydova et al., who found via simulations of a Pt/C anode (0.08 mg_Pt_ cm^–2^) that the overpotential was non-negligible at 1 A cm^–2^.^257^ Conversely, in PEMFCs, the anode operates similar to a nonpolarizable electrode.^257^

**Figure 10:**
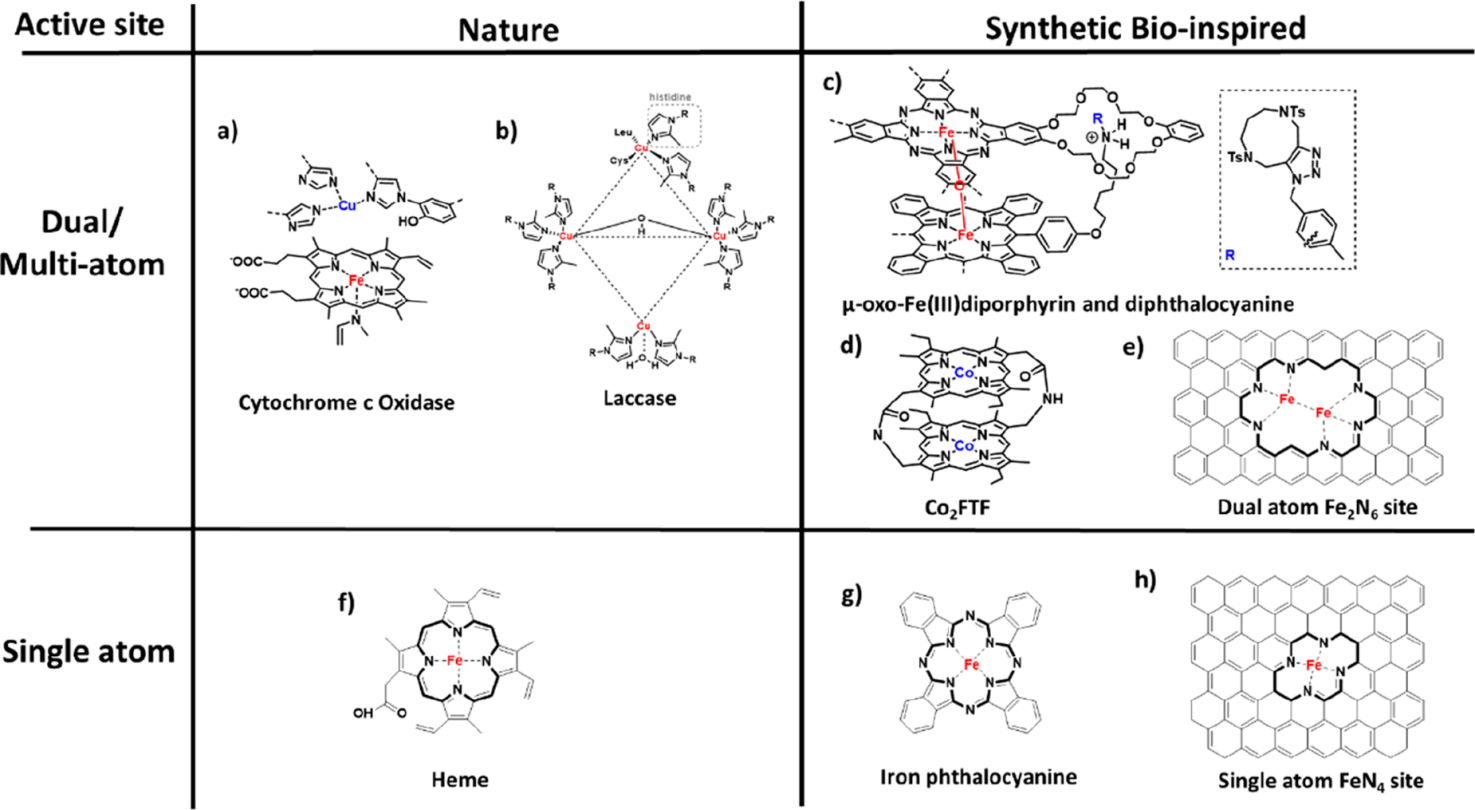
Key structural features of dual and multiatom active sites in naturally occurring (a) CcO and (b) laccase and nature-inspired M_*x*_N_*y*_ active sites in (c) μ-oxo-Fe(III)diporphyrin and diphthalocyanine. Figure 10c was adapted from ref (265). (d) Co_2_FTF. Adapted with permission from ref (266). Copyright 1980 American Chemical Society. (e) Dual-metal-atom Fe_2_N_6_ site in graphene. Key structural features of single-atom active sites in naturally occurring (f) heme and (g) iron phthalocyanine and the (h) single-atom FeN_4_ site in graphene.

To improve the activity of an ORR catalyst, two design principles can be applied: either increasing the number of active sites or enhancing the intrinsic activity per active site.^258^ Nature mastered the latter when evolving the active-site structures relevant for biochemical oxygen transport and conversion. The two main ORR enzymes are CcO, (Figure 10a),^259^ where the electron transfer of ORR drives a transmembrane proton pump, and multicopper oxidase, an ORR catalyst that concomitantly oxidizes various organic molecules and metal ions.^260,261^ The active-site structure of a multinuclear copper catalyst such as laccase is composed of four Cu atoms, which are integrated into the enzyme by a different number of amino acid ligands, mainly histidine (Figure 10b). The scientific community aims to mimic enzymes via dual atom catalysts (Figure 10c–e) or by replicating the simpler single-site catalysts derived from the heme structure (Figure 10f), the binding site for O_2_ in CcO, hemoglobin, and myoglobin, which bind O_2_ reversibly for transport.^262^ The central FeN_4_ in heme has been emulated by molecular model systems such as porphyrins, phthalocyanines, and Fe single-atom catalysts (Figure 10g and h).^263,264^

**Figure 11:**
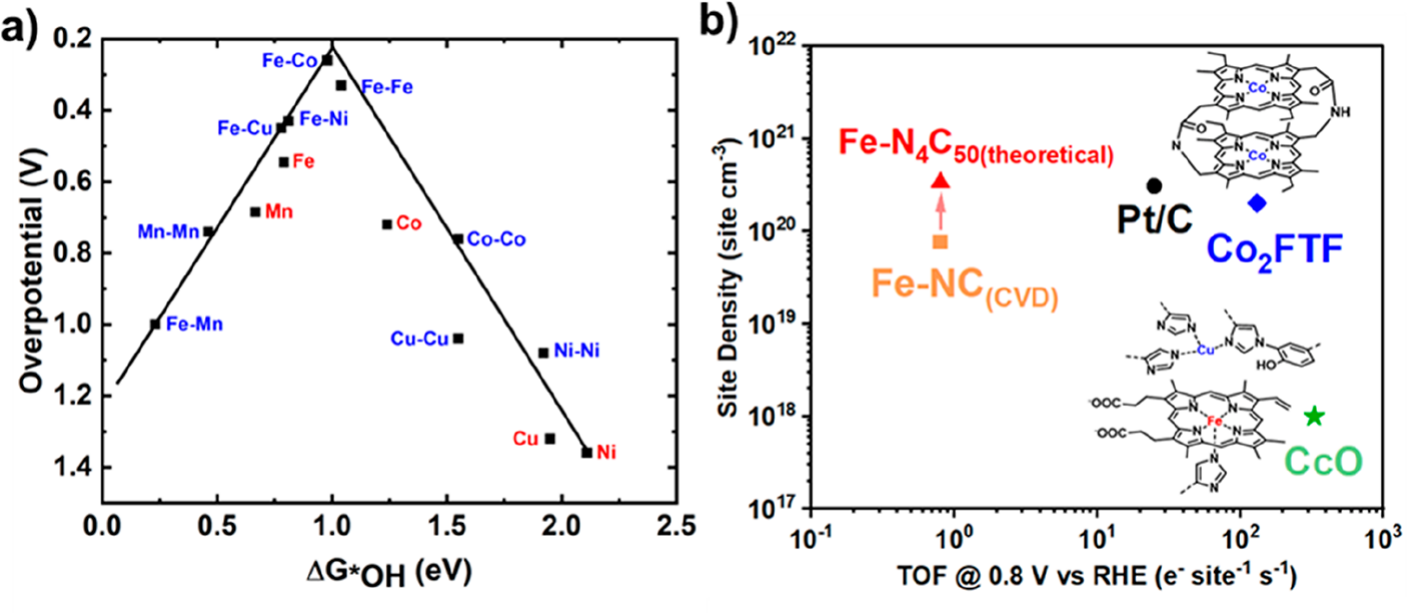
(a) Thermodynamic relations (volcano) of the overpotential for ORR calculated for single (M–N_4_–graphene, M = metal) and dual-metal-atom (M_2_–N_6_–graphene) sites versus the DFT-calculated *OH binding free energy (G_*OH_). Reproduced from ref (267). Copyright 2019 American Chemical Society. Reproduced from (276). Copyright 2019 American Chemical Society.. (b) Calculated TOF (0.8 V vs RHE) and measured (Fe-NC_(CVD)_) or calculated (rest) site density for single atoms (FeNC_CVD_ and FeN_4_C_50(theoretical)_), dual-metal atoms in molecular (Co_2_FTF) and enzyme (CcO) structures, and Pt/C. Site densities of all catalysts (excluding CcO) were calculated based on an electrode volume of 0.4 g_carbon_ cm^–3^, with Fe–N_4_C_50_ and Co_2_FTF calculated based on their molecular mass. CcO (green star) is from A. ferrooxidans CcO with a ferrocenecarboxylic acid redox mediator,^184^ with the TOF calculation provided in ref (184). The site density of CcO was calculated based on an occupied volume of ca. 1000 nm^3^ (bovine heart CcO,^277^ distances from XRD were analyzed using RCSB PDB^278^). Fe-NC_CVD_ tested in an O_2_-saturated 0.5 M H_2_SO_4_ electrolyte at 900 rpm in RDE, with a site density based on nitrite stripping and the TOF from the kinetic current density.^21^ Fe–N_4_C_50(theoretical)_ (red triangle) is based on a theoretical Fe-NC^279^ with the same TOF as Fe-NC_CVD_. The site density of Pt/C (Pt nanoparticle, black circle) was obtained from ref (253) (with Pt = 50 wt % with 25% site utilization), and the TOF was from PEM fuel-cell conditions (80 °C, 100 kPa_abs_ H_2_ and O_2_).^280,281^ Co_2_FTF (blue diamond) tested in O_2_-satured 0.5 M CF_3_CO_2_H at 250 rpm.^266^

With nature utilizing a library of earth-abundant transition metals,^68^ the choice of the active metal center for ORR has been explored both theoretically and experimentally.^264^ Computational studies of a model system with M–N_4_ sites in a graphene matrix concluded that stronger oxygen binding energies of the intermediates on Fe and Mn preferentially lead to the four-electron ORR mechanism and water as a product. In contrast, Co, Cu, and Ni, which feature weaker oxygen bonds, were found to favor the two-electron ORR toward the production of hydrogen peroxide (Figure 11a).^267^ The Fe binding energy and hence the turnover frequency (TOF) are related to the Fe(III/II) redox potential in Fe macrocycle catalysts, which can be tuned by refining the electronic structure and the coordination environment to enhance *OH binding and reach the top of the volcano.^239,268^ For instance, the electron-withdrawing ability of electronegative substituents favorably shifts the redox potential for the Fe(III/II) couple in the positive direction.^239,269,270^ Additionally, penta-coordinated transition-metal macrocycles with a fifth nitrogen ligand, reflecting the real coordination environment in the enzymatic heme sites in CcO and vitamin B12, have shown enhanced ORR activity.^271^ For instance, FePc has been anchored onto pyridine-functionalized carbon nanotubes.^272−274^ Advances in penta-coordinated transition-metal macrocycle catalysts were summarized recently.^275^

Nevertheless, single-atom active sites coordinated with nitrogen atoms embedded in graphene obey the same scaling relationships as Pt(111), causing a minimum overpotential >0.4 V (Figure 11a).^193^ Meanwhile, dual-metal-atom active sites can facilitate an optimized binding energy of each oxygen atom, allowing a reduced minimum overpotential (Figure 11a). Additionally, the 3D geometric arrangement of the active site could potentially introduce alternative reaction pathways or new types of interactions with the ORR intermediates, thereby breaking scaling relations. This 3D arrangement could be achieved with dual-metal-atom-site electrocatalysts with cofacial active sites, biomimicking CcO structures.^282,283^ The experimental evidence for diporphyrinic Co structures leading to highly selective 4e^–^ pathways has been known since 1979, following instrumental work by Collman et al.^266,284^ More recently, similar porphyrinic molecular catalysts with a cofacial binuclear active site were suggested to have the potential to circumvent the limitations of the scaling relation between *G*_OH_ and *G*_OOH_.^285^ Meanwhile, new generations of porphyrinic metal–organic frameworks (MOFs) with tailored spacing could provide another pathway to 3D dual-metal-atom sites,^286^ with structurally similar designs predicted by DFT circumventing scaling relations for ORR.^287^ What has yet to be demonstrated is a robust and conductive 3D dual-metal-atom catalyst able to withstand the harsh conditions of a fuel cell, which could potentially be provided by a new class materials termed “ordered carbonaceous frameworks”.^288,289^ Interestingly, Svane et al. modeled porphyrin-like cofacial dual-atom CoN_4_C_12_ and in-plane Co_2_N_6_/graphene sites (with O bound on the opposing side) for ORR and found that only the in-plane site results in a significant deviation from scaling relations, which could be potentially further improved by substituting the Co atoms with other metals.^290^ Compared to experimental results, synthesized in-plane dual-metal atoms of Co, determined as Co_2_N_5_ (derived from pyrolyzed Co-doped ZIF-8), have been reported to exhibit mass activity over an order of magnitude higher compared to their single-atom-site Co counterparts (at 0.75 V vs RHE).^291^ As predicted by DFT, experimentally synthesized mixed-metal dual-metal-atom catalysts, most notably those containing Fe and Co, have achieved the highest ORR activities of dual-metal atom catalysts to date,^292,293^ with some displaying stabilities of over 100 h in a fuel cell, as confirmed by postelectrochemical testing of the extended X-ray absorption fine edge structure (EXAFS) and X-ray absorption near-edge structure (XANES).^294^ Additionally, mixed-metal biomass-derived ORR catalysts containing a portion of neighboring in-plane Zn–Co dual-metal=atom sites have been produced from chitosan due to the high concentration of naturally occurring amine groups, which stabilize the metal atoms.^233,295^ However, whether biomass-derived catalysts can form dual-metal-atom sites exclusively and in a controlled manner remains to be seen. Increasing the number of reactant binding sites further to trimetal-atom^296,297^ or even multimetal-atom sites, as inspired by Laccase,^298^ can also favorably direct selective ORR to water, although controllably synthesizing mimics and stabilizing such sites for electrochemical reactions becomes even more challenging.

The theoretically predicted low overpotential of enzymes (Figure 11a) has even been demonstrated experimentally to surpass the ORR kinetics of Pt-based catalysts (and native enzymes) by modifying naturally occurring enzymes.^299^ For instance, a laccase-based enzymatic fuel cell reached considerably low overpotentials as low as 0.1 V for ORR.^300^ However, the power densities in enzymatic fuel cells fall into the 1–1000 μW cm^–2^ range,^301^ constricting their practical application.^302,303^ This is caused by the bulky protein structure, which effectively limits the number of active sites per electrode volume.^299^ As illustrated in Figure 11b, while the turnover frequency (TOF) of CcO is >300 e^–^ site^–1^ s^–1^ (at 0.8 V vs RHE), the active-site density in CcO (and other enzymes) is orders of magnitude lower compared to those in metal nanoparticles or single-atom catalysts. An ideal catalyst would maximize both TOF and the site density.^304^ Recent progress in state-of-the-art high-site-density heme-like FeN_4_ sites produced via chemical vapor deposition methods (Figure 10c, denoted Fe-NC_(CVD)_) means further possible site density improvements are limited, although possible beneficial synergistic effects could take place at the upper site-density limits. Other methods to improve the TOF may be still needed, possibly via dual-metal-atom sites. Interestingly, as shown in Figure 10c, if one could create accessible layers of enzyme-inspired active dual-metal-atom complexes, such as that of Co_2_FTF by Collman et al. (Figure 10e),^266^ it could lead to a step change in the fuel cell performance of non-PGM catalysts beyond that of PGM-based catalysts; however, these molecular complexes are typically unstable and would suffer conductivity issues.

Nevertheless, significant improvements have been made in M–NC testing and activity, the understanding of the active site, and methods of quantifying the site density, such as using probe molecules of either nitrite, carbon monoxide, or cyanide,^305−307^ which are (unsurprisingly) known to inhibit enzyme and/or heme function. In particular, remarkable progress in the performance of the heme-like active sites of Fe–NC has been achieved through collaborative consortium research efforts (ElectroCat from the U.S. Department of energy^308,309^ and PEGASUS and CRESCENDO from the EU Fuel Cells and Hydrogen Joint Undertaking^310,311^), although, like enzymes, they still possess stability over an order of magnitude below practical PEMFC stability (∼100 h vs >5000 h target).^312^ However, the commercialization of Fe–NC catalysts toward alkaline and direct methanol fuel cells is already underway.^313,314^ Focus is moving toward improving durability by understanding the mechanisms of catalysts degradation (such as demetalation, radical attack such as H_2_O_2_, active-site protonation and anion binding, and micropore flooding).^315^ Inspiration from nature’s ability to repair could be used to extend the lifetime of degraded MN_*x*_ catalysts through active-site regeneration and reactivation.^316,317^ Other bioinspired research efforts for ORR active sites should be directed toward maximizing the site density of heme-inspired FeN_*x*_ catalytic active sites and at creative ideas to break the scaling relations of ORR intermediates by engineering dual-metal-atom sites, such as those present in CcO. Future designs of catalysts could allow the local movement and flexibility of their active sites to enable optimal configurations for intermediate reaction steps. For instance, in multicopper oxidase, the Cu–Cu distance of two Cu ions decreases from ∼5^318^ to ∼3.3 Å when moving from fully reduced to fully oxidized states.^319^ In a rare example, Tanaka and co-workers have experimentally synthesized and tested for ORR a cofacial bridged μ-oxo-Fe(III) diporphyrin and diphthalocyanine connected by a flexible fourfold rotaxane dual atom catalyst (Figure 11d).^265^ Upon achieving a highly efficient and stable nature-inspired active site, macroscale reactant transport to the active site could become the rate limiting factor, the importance of which is discussed in detail in section 5.

## 4 Bioinspired and Biomass-Derived Catalysts for CO_2_ Reduction

Rising CO_2_ levels in the atmosphere have prompted scientists to prioritize their research on CO_2_ recycling through carbon capture, utilization, and sequestration processes.^320−322^ Electrochemical CO_2_ reduction (CO2RR) is one of these technologies, wherein waste CO_2_ can be converted to value-added chemicals, such as carbon monoxide, formate, methane, methanol, ethane, ethylene, ethanol, propanol, etc.^320−322^ Single-carbon products such as CO can be used for the synthesis of higher hydrocarbons with the formula C_*n*_H_2*n*+2_.^323−325^ Currently, some of the most widely utilized electrocatalysts for CO2RR are noble metals, including Au or Ag nanoparticles, that display a high selectivity toward CO,^326,327^ while others such as Ni, Fe, or Pt nanoparticles are much more selective to the competing HER. Carbon-based nanomaterials are potential alternatives to such noble-metal catalysts due to their easily available raw materials, tunable structure, chemical stability, and high electrical conductivity.^328,329^ However, pristine carbon is not active for CO2RR due to the difficulty of the first activation step to form CO_2_^–^ following the reaction
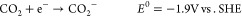


This is due to stable linear structure of CO_2_, which translates into a large thermodynamic barrier of −1.9 V vs SHE. Thus, pristine carbon remains inert toward CO_2_ activation due to its symmetric charge distribution.^329^ Heteroatom (such as boron, nitrogen, oxygen, phosphorus, or sulfur) doping resolves this issue through redistributing the charge density of the carbon matrix.^330−332^ Positively charged carbon atoms generated due to heteroatom doping can promote the adsorption of CO_2_ and the stabilization of the intermediate, thereby lowering the CO_2_ activation energy.^328^ Furthermore, porous and tunable carbon nanostructures enhance the local concentration of CO_2_, thus favoring CO_2_ reduction. It was recently observed that nonmetal dopants such as nitrogen, sulfur, and oxygen have minimal contributions to hydrogen evolution, while metal dopants in amounts above 100 ppm promote hydrogen evolution.^333^ Thus, the utmost care should be taken to prevent metal contamination from the electrolyte or the electrochemical cell. In addition to the doping, the morphology of the nanostructure also plays a crucial role. A nanoconfinement effect induced by the porous carbon nanostructures enhances the concentration of CO_2_ near the electrode surface, thus promoting the adsorption of CO2RR intermediates and favoring C–C coupling.^334^

CO2RR to different products such as HCOOH, CH_3_OH, and CH_4_ involves a multiple proton–electron transfer process (as described in reactions below) and thus faces significant challenges such as (i) low current density, (ii) unsatisfactory product selectivity, (iii) low catalyst stability, and (iv) competing HER.
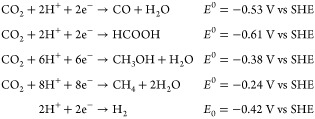


Most of these challenges can be resolved by means of bioinspired approaches through either mimicking the active sites of a known enzyme or replicating the features of the secondary enzyme that protects the active site. Therefore, in this section we delve into the recent developments of bioinspired catalysts for CO_2_ reduction and how emulating natural enzymes in a solid heterogeneous catalyst can lead to the sustainable production of high-added-value chemicals from such electrochemical process.

The presence of diverse active sites (vacancies, defects, grain boundaries, undercoordinated edges, and step sites, among others) on a polycrystalline metal catalyst, such as Cu, is responsible for lower product selectivity.^335^ Such sites have different intermediate adsorption energies and hence result in the formation of a wide array of products, such as CO, CH_4_, C_2_H_4_, C_2_H_5_OH, etc.^336^ It is thus imperative to design electrocatalysts with uniform active sites to improve product selectivity and circumvent expensive purification processes, such as reverse osmosis, electrodialysis, etc.^337^ For instance, using technoeconomic analysis, Zhu et al. reported the production cost of formic acid to be ∼0.2 USD kg^–1^, which is significantly cheaper than the current market price of ∼0.7 USD kg^–1^.^338^ When purification steps are considered, the cost increases to ∼1 USD kg^–1^, rendering the process commercially unviable. However, this issue can be resolved with enhanced catalytic performance and selectivity.

Enzyme-inspired catalytic materials with atomically dispersed metal sites can inspire us to foster the large-scale sustainable production of value-added products with better selectivity.^339^ Natural metalloenzymes such as formate dehydrogenases (found in bacteria and yeast) use a Mo- or W-based mononuclear metal complex to reduce CO_2_ and produce HCO_2_H.^340^ Molecular catalysts that imitate such active sites have been reported, though none were active toward CO2RR.^341^ CO dehydrogenases (CODHs), found in aerobic and anaerobic bacteria, catalyze the reversible transformation of CO_2_ to CO using either a Mo–S–Cu active site (Mo–Cu CODH)) or a Ni center bound to a unique Fe_4_S_4_ cluster (Ni–Fe CODH) polynuclear complex.^342,343^ Mougel et al. synthesized a [(bdt)Mo^VI^(O)S_2_Cu^I^CN]^2–^ (bdt = benzenedithiolate) bimetallic molecular complex, inspired by Mo–Cu–CODH, that successfully catalyzed CO_2_ reduction to formate with a FE of 74% at −2.37 V vs Fc/Fc^+^.^344^ However, CO_2_ was instead reduced to CH_4_ (FE_CH4_ = 12%) on the surface of another Ni–Fe-based molecular catalyst inspired by Ni–Fe–CODH active sites.^344^ This difference in the selectivity pattern of molecular complexes could be attributed to the lack of secondary features compared to their enzymatic counterparts. Such features, mostly peptide matrices,^345^ are critical for stabilizing reaction intermediates, shuttling reactants between active sites, introducing hydrophobicity, and removing products from the active sites in order to enhance efficiency and selectivity (as discussed in section 5).^346^ Molecular catalysts are indeed advantageous in terms of mimicking the active sites of enzymes. Owing to their well-defined active sites, mechanistic studies and structure–activity correlations can be monitored easily.^347,348^ However, such catalysts possess low electronic conductivity and stability; hence, it is desirable to either support them on a conducting substrate prior to electrocatalysis or subject them to high-temperature pyrolysis to enhance the conductivity, although this leads to the aggregation of metal centers, the degradation of the well-defined active sites and undesirable side reactions such as carbothermal reduction.

**Figure 12:**
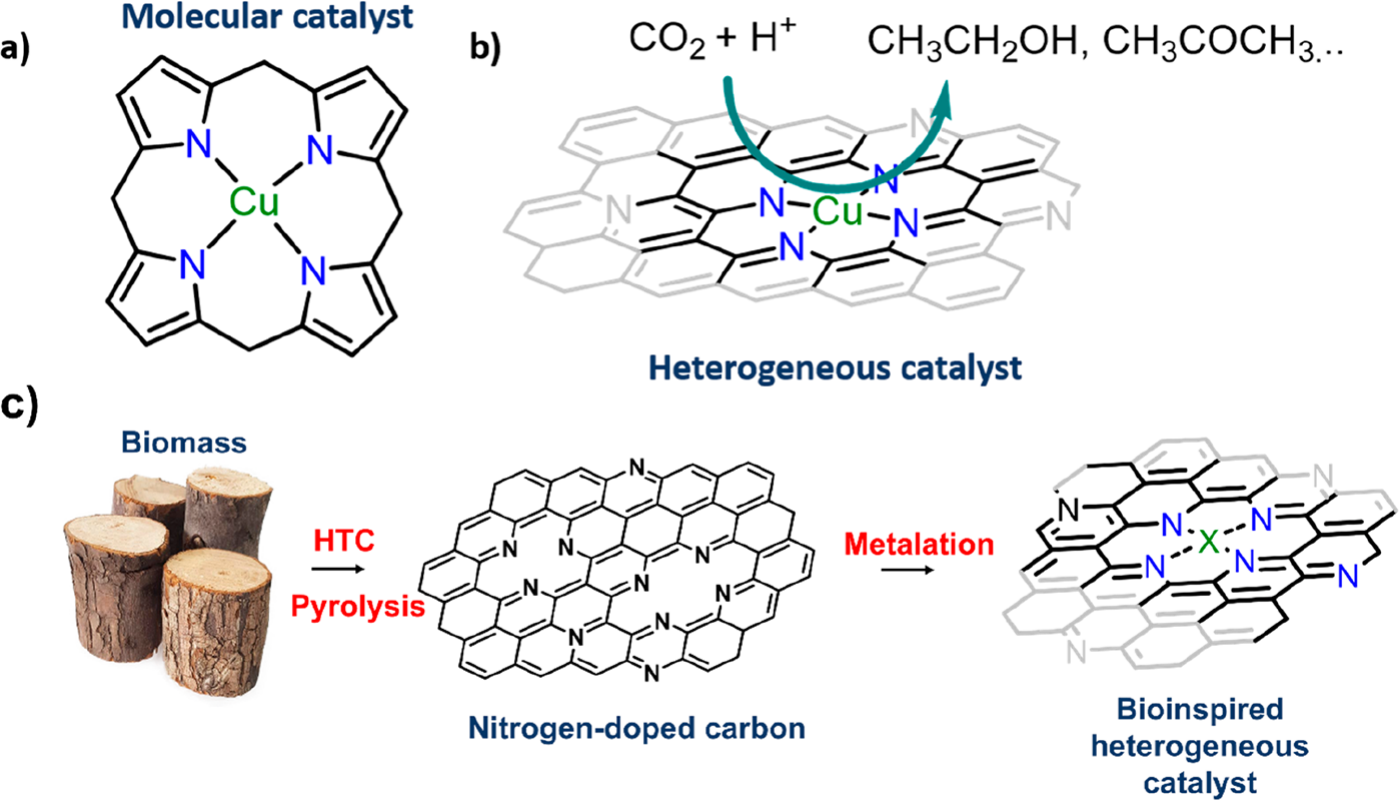
(a) Cu–porphyrin, (b) CO_2_ reduction over CuN_4_ sites embedded in a carbon matrix, and (c) schematic representation of the synthesis of bioinspired heterogeneous catalysts from biomass.

Porphyrins and phthalocyanines are another group of heterocyclic organic compounds that are found in a wide range of enzymes and can activate small molecules. The implementation of their active site embedded within carbon matrixes has been widely explored in electrocatalysis such as ORR (as discussed in section 3). In CO2RR, these materials are typically selective toward the formation of CO.^349−352^ Another major challenge in CO2RR is the lower selectivity for the formation of C_2+_ products, which are commercially more valuable than C_1_ products. Competition between C–C, H–H, and C–H bond formation makes it difficult to produce multicarbon products during CO2RR in an aqueous environment.^353^ Metallic Cu and oxides favor the formation of large-chain hydrocarbons due to the strong CO adsorption, allowing subsequent C–C coupling.^335^ Nevertheless, they do not allow maximum atom utilization, and it is still a challenge to modulate C–C coupling and obtain high faradaic efficiencies for long-chain hydrocarbons.^335^ Therefore, while supporting Cu nanoparticles in N-doped supports can lead to the production of C_2_ products in a moderate yield (FE_ethanol_ = 63% at −1.2 V vs RHE),^354,355^ achieving maximum atom-utilization efficiency through the formation of Cu–N_4_ sites within a carbon matrix has emerged as a very effective alternative to achieve C_2+_ products with high faradaic efficiencies (Figure 12a and b). Fontecave and co-workers recently showed the production of ethanol with a 55% faradaic efficiency over CuN_4_ single sites in 0.1 M CsHCO_3_, although the strong reducing potential of −1.2 V degraded them into Cu nanoparticles, which are undetectable using the ex-situ techniques.^356^ Utilizing similar materials, Zhao et al. reduced CO_2_ to acetone with a faradaic efficiency of 36.7% in 0.1 M KHCO_3_ at −0.36 V, with the Cu–pyrrolic N_4_ sites acting as the active centers for C–C coupling.^357^ While these results are encouraging, no report has successfully replicated monoxide dehydrogenase-type Cu dual-atom catalytic sites, which would promote bridge-type adsorption and break the scaling relationship.^358^ There have been a number of recent reports of the computational screenings of dual-atom catalysts for CO2RR. Wan et al. modeled a cofacial diporphyrin-based 3D electrocatalyst with a dual-metal-atom center (Co–Co) that was computationally predicted to produce hydrocarbon products from CO_2_. Such a site can stabilize reaction intermediates such as *CH_2_O, *OCH_3_, and *OCCHOH, resulting in the production of multicarbon products; however, the stability of such catalysts would be a large challenge.^359^ Zhao et al. studied the CO2RR performance of Cu dual-atom catalysts supported on C_2_N materials and observed that CH_4_ and C_2_H_4_ were the main obtained products due to the moderate binding energies for the reaction intermediates HCOO* and HCOOH*.^360^ Unlike Cu–N_4_ materials, which tend to degrade into Cu NPs at negative potentials, Fe-NC catalysts have been proven to be more robust under CO2RR conditions. Gu et al. showed the performance of an Fe^3+^–NC material with pyrrolic coordination for the reduction of CO_2_ to CO.^361^ Besides the remarkable performance (>90% faradaic efficiency at −0.45 V vs RHE), these catalysts were shown to remain stable at potentials up to −0.5 V vs RHE, where Fe^3+^ started to be reduced to Fe^2+^ and the pyrrolic coordination started to degrade. Achieving such stability in an Fe-based dual-atom catalyst, which provides bridge-mode adsorption,^362^ could potentially lead to the bioinspired large-scale production of C_2+_ chemicals.^363^ Indeed, Chan and co-workers computationally screened a wide variety of Fe–M combinations supported in nitrogen-doped carbons using a potential-dependent microkinetic model based on CO_2_* and COOH* binding energies as activity descriptors. Such dimers exhibit a two-site bidentate binding mode to the reaction intermediates that results in surface dipoles, promoting the CO_2_-to-CO activity (comparable to that of Au (211)) and decreasing the selectivity for HER.^364^

Several reports of biomass-derived nitrogen-doped carbons being used as CO2RR catalysts have also been published; however this approach is still at its infancy. Huang and co-workers demonstrated the synthesis of defective N-doped carbon derived from a silk cocoon, but the absence of a metal resulted in moderate currents (<5 mA cm^–2^) and selectivity toward CO (FE_CO_ = 89%).^365^ Combining biomass-derived nitrogen-doped carbons with an appropriate method of controlled metalation can potentially lead to the formation of a new generation of sustainable, highly active materials to produce value-added chemicals (Figure 12c).^244^

## 5 Bioinspired Interfaces and Mass Transport

As discussed above, the active sites of enzymes have been mimicked in molecular catalysts for the carbon dioxide reduction reaction, oxygen evolution, and ORR. However, chemical reactions carried out by enzymes are typically much more efficient and selective than their manmade counterparts. This suggests that the complex protein matrix surrounding the active sites in enzymes has a significant impact on the activity, specificity, and even durability of the enzyme.^366^ One of the most essential roles of this outer coordination sphere is the spatial and temporal control of reactants and products delivered to the active site.^366^ For example, CcO offers a defined path for the transport of oxygen, water, protons, and electrons. At the same time, the environment around the active site is finely tuned to control the reactivity and specificity of the active site itself.^259^ On the other side, the reaction in electrochemical cells happens at the triple-point interface between the liquid electrolyte, the gaseous reactant or product, and the solid catalyst, with no defined pathways for the reactants and products. This causes significant mass-transport limitations, particularly for the case of nonprecious metal and bioderived catalysts, which require higher loadings compared to their precious-metal counterparts.

To improve transport and accessibility to catalyst sites, researchers have once again taken inspiration from nature, creating bioinspired 3D supports with hierarchical pore size distributions by designing dedicated proton channels similar to those present in enzymes. Finally, they have optimized water and bubble management, taking inspiration from natural hydrophobic and hydrophilic surfaces.

### 5.1 Catalyst Nanostructures

Catalyst structure development has long benefited from bioinspiration in terms of surface area and site density, for instance, by directly employing biomass in catalysts synthesis with the aim to retain the structural features in the resulting materials. Nevertheless, catalysts derived directly from biomass could suffer from inconsistencies across samples, limited supply, and competition with food resources. Therefore, attempts have also been made to mimic the natural structure with other scalable and accessible materials. In terms of OER electrocatalysts, there have been various bioderived carbon allotropes featuring three-dimensional and hierarchical structures reported to facilitate charge and mass transport; for example, a 3D cotton-derived N-doped carbon microtube^113^ possesses interconnected hollow graphitized fibers, and plant-derived porous carbon inherits a tubular array structure with a wide range of macro- to mesoporosities,^367^ building multilevel transporting channels. Carbon networks delivering effective conductivity and physical sturdiness can excellently accommodate metallic compounds or nanoparticles for synergetic catalytic effects.^121,368−373^ In such cases, although metallic compounds are the main OER catalysts, the carbon support plays a critical role in dispersing and stabilizing metal-based nanoparticles within its skeleton, affording accessible active sites, ensuring rapid charge transfer at the interface, and facilitating gas diffusion throughout the bulk. For example, Guan et al. observed the superior catalytic activity of CO_3_O_4_ nanoparticles anchored on cattle-bone-derived nitrogen-doped carbon. The activity was attributed to the uniform distribution of Co_3_O_4_ on the carbon matrix, the large specific surface area (1070 m^2^ g^–1^), and the well-defined porous network of the carbon framework.^61^

When looking at improving mass transport in ORR, Liu et al., inspired by the shape of a grape cluster, integrated 1-D nanofibers with isolated carbon spheres to obtain a 3D framework with enhanced electron and mass transfer by combining the electrospinning strategy with the in situ growth of polydopamine.^374^ Similarly, unique structures such s honeycomb or pomegranate have inspired many works on the development of semblable catalyst structures. In particular, the honeycomb structure features mechanical stability from the hexagonal channels along with highly ordered pores. Wang et al. prepared N,P-codoped honeycomb-shaped carbon nanoarchitectures via the hydrothermal treatment of melamine, phytic acid ,and glucose, followed by freeze-drying and pyrolysis carbonization. The porous structure featured highly aligned and interconnected open macrochannels, which provided multiple diffusion paths for the electrolyte and facilitated mass transportation within the catalyst material.^211^ Meanwhile, inspired by the stomata structure, Han et al. fabricated 2D Cu–N–C nanodisks with an interconnected hierarchical porous topology from Cu-containing MOFs. The stomata-like hierarchical porous structure possessed more exposed Cu single-atom sites exhibiting ORR performance comparable to that of commercial Pt/C catalysts, which was much higher than the Cu–N–C structures without this structural feature.^375^

**Figure 13:**
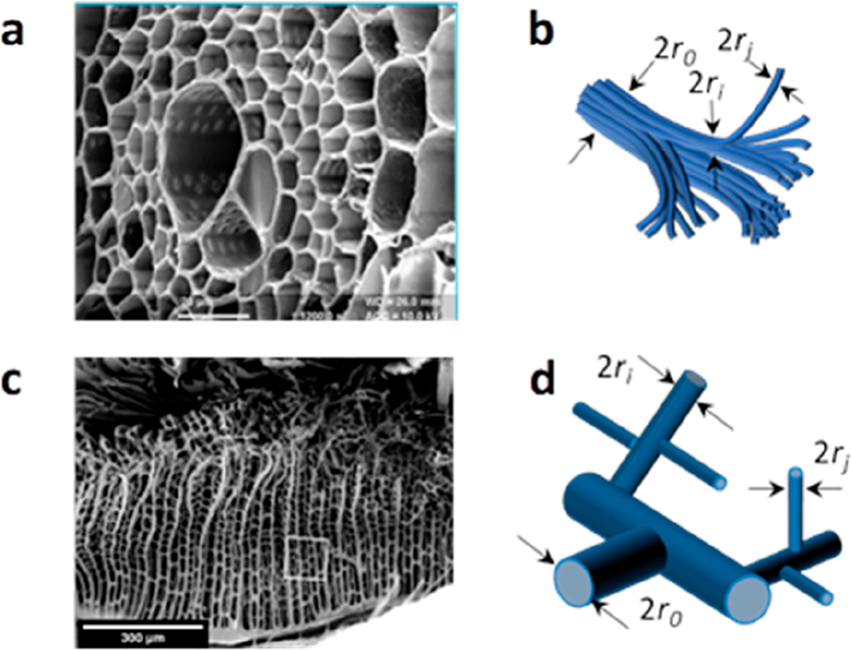
Hierarchically porous structures of living Murray networks in a leaf.^380^ (b) Schematic illustration of the branch network in a leaf. (c) Hierarchically porous structures of living Murray networks an insect.^381^ (d) Schematic illustration of the branch network in an insert. Panel (a) reproduced from ref (380). Copyright 2017 Wiley publishing group. Panel (b) and (d) adapted from ref (382). Copyright 2017 Springer Nature. Panel (c) reproduced from ref (381). Copyright 2001 The Company of Biologists Limited.

**Figure 14:**
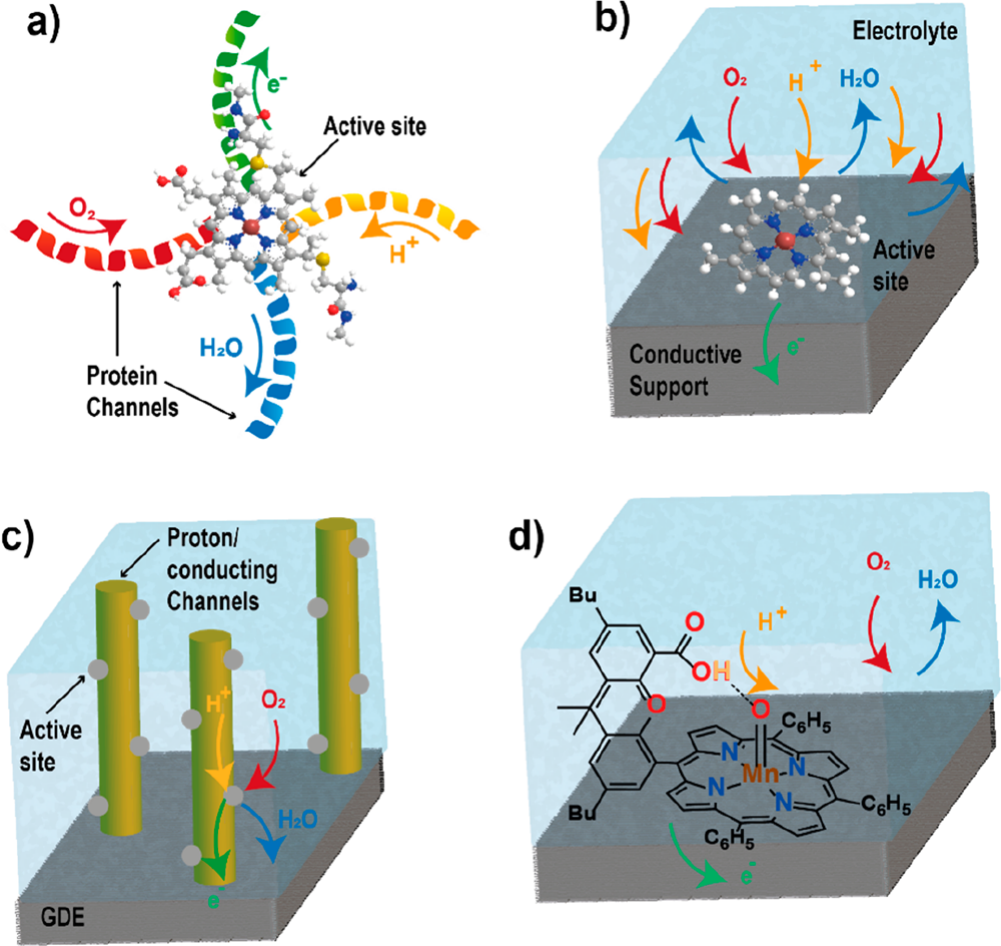
(a) Schematic of the enzyme CcO showing the FeN_4_ active site surrounded by the outer coordination sphere, which features defined channels for the transport of oxygen, water, protons, and electrons. (b) Schematic of molecular catalysts showing the active site deposited on a conductive support. In this configuration, the reaction happens at the triple-point interface between the aqueous electrolyte, the solid catalyst, and the gaseous reactant, with water, oxygen, and protons transported through the electrolyte together. (c) Schematic of the enzyme-inspired architecture proposed by Xia et al.,^76^ where protons and electrons are transported to the active site via ordered proton-conducting and electron-transporting channels on which the active site (Pt nanoparticles) has been deposited. (d) Schematic of a “Hangman” porphyrin featuring an acid–base group above the porphyrin macrocycle, which imitates the structure and functionality of the amino residues in the distal cavities of heme hydroperoxidase and offers a defined pathway for proton transport.

Indeed, many works have reported enhanced ORR performance as a result of the hierarchical structure, and some of them have focused on understanding the different roles of micro-, meso-, and macropores in ORR.^376,377^ Nevertheless one of the main remaining challenges is to control the pore distribution of biomass-derived materials by the predictive design of a hierarchical structure. Back in 1926, Murray reported the secret of the vascular network in terms of minimizing transport costs (Murray’s Law), which can serve as a powerful biomimetics design tool.^378^ This principle is based on the hierarchical porous structures of different organisms, where the pore dimensions decreased steadily across multiscale to achieve a maximized transport ability. This is done in different ways in nature depending on whether the organisms require liquid transportation or gas diffusion. For instance, in leaf veins where one parent branch splits into *n* child branches (Figure 13a and b), the law states that
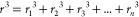
where *r* is the radius of the parent branch and *r*_1_, *r*_2_, ..., *r*_*n*_ are the radii of child branches. The constant pore volumes at different scales guarantees optimized flow. In contrast, insects rely on surface gas exchange for breathing. here, the sums of the surface area remain the same rather than the volume to maximize the delivery of gas components (Figure 14c and d),^379^ and the law states that^379^
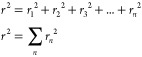


However, Murray’s law was completely overlooked in chemistry and materials science until 2017 when Su et al. revisited it and both provided a generalized equation to correlate the micro-, meso-, and macropores and successfully developed a series of “Murray materials” based on it.^382^ A microporous ZnO nanocrystal was used as the primary building block to construct a hierarchically porous network layer-by-layer through evaporation-driven self-assembly. The resulting Murray materials revealed excellent interconnected channels ranging from micro- to meso- to macr-scale and demonstrated significantly improved mass transfer in a three-phase electrochemical reaction. Nevertheless, the tedious synthesis process hindered its industrial application to general material preparation and resulted in very limited following up work.^383,384^ Following this work, several attempts have been made to prepare Murray-type assemblies via less demanding synthesis processes, for example, through a simple pyrolysis method leading to a Murray-type assembly of Co–NC nanoparticles with a multiscale intraparticle porous network.^385^ These materials demonstrated activity for ORR comparable to that of commercial Pt/C in alkaline conditions, but further insights are required to confirm whether the exhibited hierarchical structure truly follows Murray’s Law.

In summary, the advancement of the development of fuel cell catalysts has long benefited from nature on aspects ranging from active sites imitating CcO and heme to electrocatalyst structures replicating hierarchical natural networks. Researchers have learned a lot from nature in terms of structure engineering to increase accessible, high-site-density catalysts. This can be done via (1) directly employing existing three-dimensional biomass to prepare the catalysts, (2) imitating the specific structure or properties of a given biosystem, or (3) developing a hierarchical structure inspired by nature. However, significant challenges remain in two main aspects: (1) theoretically understanding the ideal pore features for ORR to clarify the requirements for catalyst structures with predictive design and rational synthesis and (2) developing more sustainable and effective catalyst preparation methods to avoid the complex and costly template-involved process. Overcoming these two challenges will help maximize the utilized site density for high-performance bioinspired ORR catalysts. Nature offers much ahead of our current technology, especially in terms of the capability of systematically fulfilling all objectives at different scales. Therefore, one can expect that fuel cell catalysts of the future will feature many aspects taken from nature.

### 5.2 Proton Conduction

Fuel cells and electrolyzers necessitate either proton or hydroxide conduction, depending on the pH. Since acidic conditions are the most common and well-developed for both technologies, this section will focus solely on bioinspired proton conduction, which is also a common requirement for CO_2_ and N_2_ reduction cells. Lack of access to protons can reduce catalyst utilization for all the above-mentioned reactions, and limited proton conduction in the catalyst layer can become rate-determining, limiting the overall performance of the catalyst. Proton transport is usually achieved by physically mixing the electrocatalysts with the polymer electrolyte Nafion, which also acts as a binder for the catalyst layer. Despite its outstanding proton conductivity and excellent chemical stability, Nafion suffers from high costs and limited performance at high temperatures. Additionally, the random proton carrier distribution obtained by drop-casting the catalyst ink with physically mixed Nafion rarely matches that of the catalytic sites, leading to an inefficient proton -transfer network. The high molecular weight of Nafion also precludes it from accessing the smallest pores, exacerbating the initial problem and further reducing catalyst utilization. Furthermore, in fuel cells, the transport of protons competes with that of water and oxygen, which all diffuse in the liquid electrolyte (Figure 14b). This is a very different scenario from what happens in enzymes, which rely on specific pathways for the spatial and temporal control of reactant and product delivery. In the case of cytochrome-c, this consists of separate channels for the transport of oxygen, water, protons, and electrons (Figure 14a). Nonpolar oxygen is delivered to the active site through a hydrophobic channel, while produced water is transported away to the Mg site within milliseconds of the reaction with oxygen.^259^ Electrons are transported long-range via buried metal centers bound to the polymer matrix, while protons move in dedicated proton channels wherein the proton is free and moves by hopping from an oxygen ion to another, breaking and reforming covalent and hydrogen bonds via the so-called by means of the Grotthuss mechanism. Mimicking these structures, researchers have proposed engineered catalyst layer designs with dedicated pathways for the transport of protons.

Taking a bioinspired approach, Pillai and co-workers mixed Nafion with plant hormones as low-molecular-weight proton conductors. They reported that indole-3-acetic acid significantly increased the electrocatalytic surface area of the Pt catalyst used (from 30% to 60%) and improved the fuel cell performance by 150 mW cm^–2^.^386^ Even though it was demonstrated for Pt-catalyzed ORR, this approach could be beneficial for all the electrocatalytic reactions that rely on access to protons. In an attempt to mimic the outer coordination spheres of enzymes, Xia et al. synthesized a hierarchically ordered structure with rationally designed channels for proton transport (Figure 14c).^76^ This architecture was obtained by electrochemically polymerizing pyrrole decorated with Nafion ionomers directly on a gas diffusion layer, thus obtaining ordered proton-conducting channels. Consequently, platinum Pt nanoparticles were decorated on the so-obtained arrays. Using this enzyme-mimicking, the authors obtained a specific power density of 5.23 W mg^–1^_Pt_, 3.7× that obtained with a commercial catalyst coating.^76^

An additional approach to mimic proton transport in enzymes consists of the use of Hangman prophyrins, which provide a distinct pathway for protons to reach the metal center and the active site of the single-atom catalyst. These molecules poise an acid–base group above the porphyrin macrocycles, capturing the structure and functionality of the amino residues in the distal cavities of heme hydroperoxidase (Figure 14d).^387^ In the case of electrocatalysis, this architecture defines a pathway for the transport of proton to the metal center present on the porphyrin, and in the case of ORR it promotes selectivity toward the 4e^–^ pathway.^388,389^ In addition, other distal residues, which can form hydrogen bonds to bound oxygen species at the active Fe site of the porphyrin, have been shown to affect the ORR rate by a pH-dependent “push” and “pull” effect.^390,391^ At low pH, the pendant residues are protonated, stabilizing the Fe^III^–OOH species and facilitating the cleavage of the O–O bond in a “pull” effect similar to that observed in peroxidase. In contrast, at higher pH, a “push” effect characteristic of cytochrome P450 is observed by which the trans-axial water is deprotonated to a hydroxide, increasing the p*K*_a_ of Fe^III^–OOH.

Proton transport, and more specifically ionomer distribution and utilization, in the catalyst is still a much-overlooked problem. Despite the growing interest regarding Nafion replacement in proton-exchange membranes, very little research has been directed toward alternative ionomers for the catalyst layer. Nevertheless, the limited examples reported above show how enzyme-mimicking could pave the way for the synthesis of improved catalysts, for example, by developing defined channels for the transport of protons similar to those present in enzymes or by mimicking the “push” effect characteristic of cytochrome P450 with distal residues.

### 5.3 Water and Bubble Management

**Figure 15:**
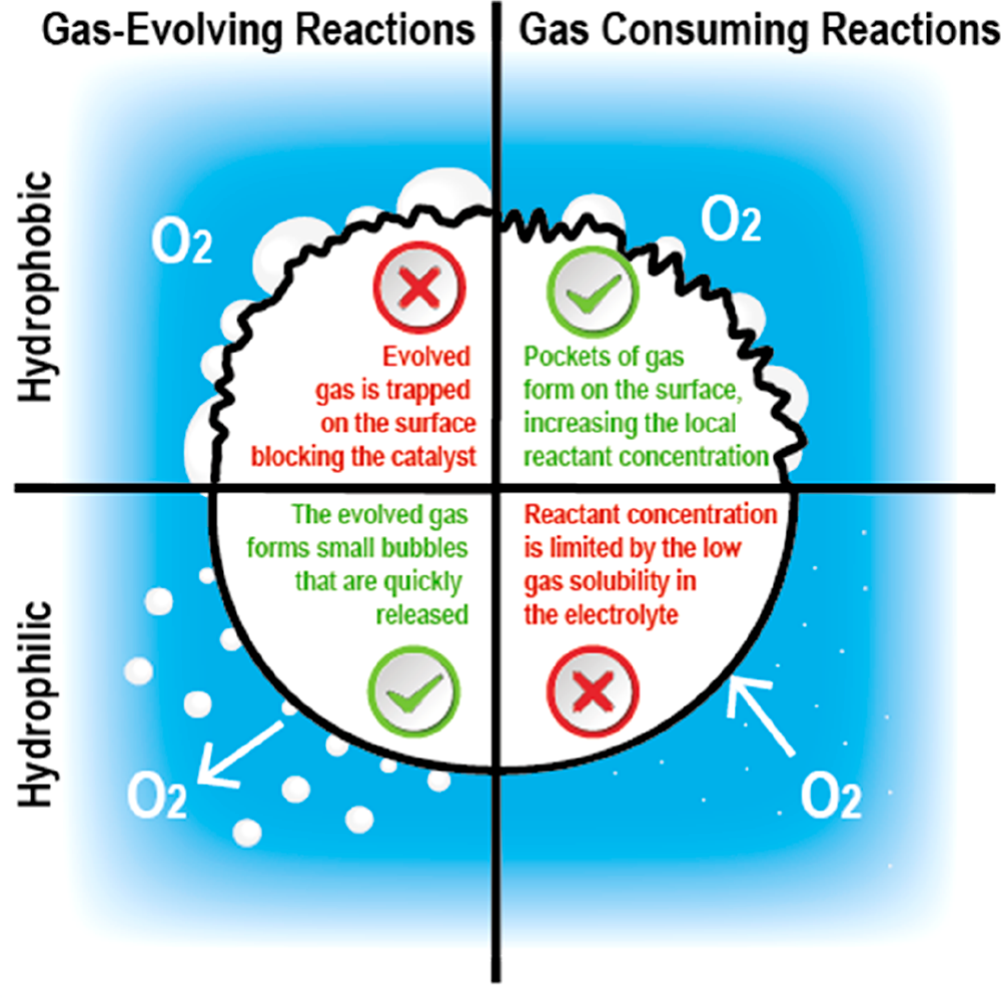
Schematic showing the effects of hydrophobic and hydrophilic surfaces on reactions evolving gases (such as OER and HER) and consuming gases (such as ORR, HOR, and CO_2_RR).

As discussed above, enzymes benefit from a defined pathway for water and gas transport. These structures are currently too complex to be reproduced in an electrocatalyst, but the electrode surface can be modified to tune the hydrophobicity at the triple-point interface, hence controlling gas and water access at the surface (Figure 15).

Nature offers several examples of how chemical composition and hierarchical structures can be employed to effectively manipulate hydrophobicity and gas bubble behavior, and several comprehensive reviews have been published on the topic^74,392−395^ without discussing the application of these modifications to electrocatalysis, which will be the focus of this section. For example, lotus leaves feature micropapillae and nanobranch-like hydrophobic wax crystals that lead to a superhydrophobic surface with excellent bubble-bursting performance.^396^ On the contrary, *Salvinia* offers long-term air retention thanks to its hydrophilic pins and hydrophobic pedestals.^397^ Electrochemical reactions have a wide range of requirements in terms of aerophilicity, which is mainly dependent on whether the gas is evolved or reacted. The required properties have been achieved by bioinspired electrodes with tuned chemical compositions and microstructures.

**Figure 16:**
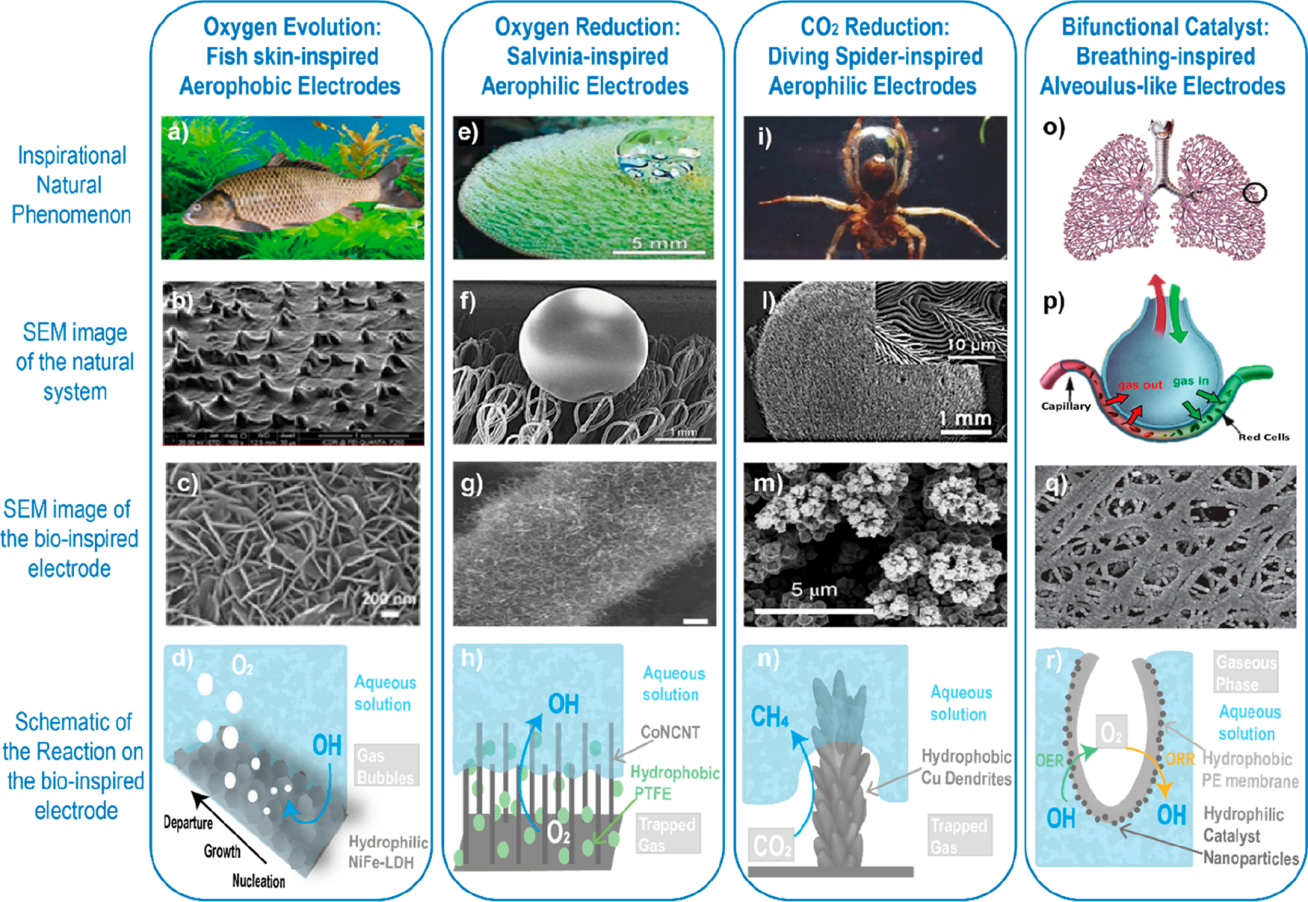
Aerophobicity for oxygen evolution: (a) Picture of a carp and (b) scanning electron microscopy (SEM) image of a fish scale skin showing underwater superaerophobicity. Reproduced with permission from ref (398). Copyright 2017 American Chemical Society. (c) SEM image of a fish-scale-like aerophobic NiFe-LDH electrode. Reproduced with permission from ref (399). Copyright 2016 WILEY-VCH Verlag GmbH & Co. KGaA. (d) Schematic of oxygen evolution on a fish-scale-like aerophobic electrode, which helps the release of evolved gas bubbles. Aerophilicity for ORR: (e) picture and (f) SEM image of floating water fern *Salvinia*. The egg-beater-shaped hair array on the salvinia surface consists of hydrophilic pins and hydrophobic pedestals that allow it to trap air. Reproduced with permission from ref (397). Copyright 2010 WILEY-VCH Verlag GmbH & Co. KGaA, Weinheim. (g) SEM image of Co nitrogen-doped carbon nanotubes on carbon fiber paper. Reproduced with permission from ref (400) Copyright 2016 WILEY-VCH Verlag GmbH & Co. KGaA. (h) Schematic of CoNCNT with hydrophobic PTFE nanoparticles.^397^ This aerophilic electrode creates a layer of trapped oxygen, increasing the catalytic activity. Aerophilicity for CO_2_ reduction: (i) Picture of a water spider and (l) SEM image of its skin. The feather-like hair on its abdomen allows the water spider to efficiently trap air bubbles underwater. (i) reproduced with permission from ref (402). Copyright 2013 Neumann and Woermann; licensee Springer. (l) reproduced with permission from ref (401). Copyright 2011 The Company of Biologists. (m) SEM image of the water spider-inspired hydrophobic Cu dendrite. Reproduced with permission from ref (73). Copyright 2019 Springer Nature. (n) Schematic of CO_2_ reduction on the hydrophobic Cu dendrite. The formation of a layer of trapped gas increases the local CO_2_ concentration, improving the selectivity toward CO_2_ reduction rather than hydrogen evolution. Bifunctional OER and ORR catalysts: (o and p) Schematics of the breathing process in alveoli. Reproduced with permission from ref (75). Copyright 2018 Elsevier Inc. (q) SEM image of a PE membrane modified with a Ag/Pt catalyst. Reproduced with permission form ref (75) Copyright 2018 Elsevier Inc. (r) Schematic of the alveolus-like structure of the catalyst-modified PE membrane, presenting a hydrophilic side in contact with the electrolyte and a hydrophobic side in contact with the gas.

For all the gas evolution reactions, such as HER and OER, hydrophilic surfaces are preferred (Figure 16). Gas products can adhere on hydrophobic catalysts, forming a continuous film and blocking the electrolyte’s access to the active site. In contrast, hydrophilic surfaces reduce the size of gas bubbles and their time of residence on the catalyst, leading to stable electrochemical behavior and higher catalyst utilization.^403^

Kim et al. synthesized nickel phosphorus films with identical chemical compositions and tuned their contact angles with different complexing agents. The so-synthesized electrodes were used as HER catalysts, demonstrating that increasingly hydrophilic surfaces provide superior performance; specifically, the contact angle reduction from 77° to 40° caused a 134 mV reduction in overpotential at 100 mAcm^–2^ current density.^398^ Similar beneficial effects of hydrophilic electrodes have been reported for hydrogen^399,404−406^ and oxygen evolution.^399,406−408^

A further increase in hydrophilicity can be obtained via bioinspiration. Fish skin displays superaerophobic properties that reduce gas bubbles on their bodies, thus improving their balance and resistance to swimming. The skin of most fish is composed of fan-like scales, coated in hydrophilic mucus, and covered in ordered micropapillae (Figure 16 a and b). A similar structure was obtained by Xu et al., who used a one-step hydrothermal process to synthesize NiFe-layered double hydroxide nanoplates offering a gas contact angle of 150° (Figure 16c).^399^ The as-synthesized superhydrophilic material offered exceptional oxygen evolution performance and fast gas product release with an average bubble size of 39 μm, compared to the impeded release of 220 μm gas bubbles observed for IrO_2_/C (Figure 16d).^399^

Meanwhile, ORR relies on access to the gaseous reactant and rather benefits from hydrophobic surfaces (Figure 16e).^400,409−411^ This was demonstrated by Sun and co-workers, who obtained a superhydrophobic surface by modifying a carbon paper with poly(tetrafluoroethylene) nanoparticles to yield a stable oxygen gas layer underneath the cobalt-incorporated NCNT catalyst, which displayed superior performance in both acidic and alkaline media (Figure 16 g and h).^400^ This architecture resembles that of floating water fern *Salvinia molesta* which, thanks to its eggbeater-shaped structure, can hold air underwater for several weeks (Figure 16e and f).^397^ Another approach recently introduced to control the presence of water and gas at the electrochemical interface is the use of ionic liquid layers. Oxygenophilic and hydrophobic ionic liquids, such as those used as coatings on the surface of electrocatalysts, can modify the triple-point interface, improving oxygen concentration and water expulsion. A recent review by our group has summarized the unexplored potential of ionic liquid layers.^412^

Hydrophobicity also has a positive, and perhaps an even more drastic, effect on CO_2_ reduction due to (1) the low solubility of CO_2_ (33 mM) in aqueous electrolytes causing a mass-diffusion limitation for CO_2_ to the electrode surface^413^ and (2) the competition with the more thermodynamically favorable HER. In nature, hydrophobicity is utilized to solve these issues; for example, active sites in enzymes such as CODHs/acetyl-CoA synthase are protected by the presence of a hydrophobic protein framework.^413^ Hydrophobic secondary features close to the active sites create CO_2_ gas pockets, thus suppressing the competing hydrogen evolution and enhancing the conversion rate of CO_2_ to glucose. Thus, a bioinspired cathode can be designed with a catalyst on one side and a hydrophobic polymer on its opposite side, imitating the role of a metalloenzyme.^414^

Mougel and co-workers have taken inspiration from another natural example, namely, diving spiders, which make use of the plastron effect to breath underwater. The plastrons are composed of hydrophobic feather-like hairs, which present both nanoscale and microscale surface structures able to trap air underwater (Figure 16I and j). They achieved a similar multiscale surface by modifying a hierarchically structured Cu dendrite, with a monolayer of waxy alkanethiol (Figure 16m and n). As a result, the hydrophobic electrode can trap a thin film of CO_2_, increasing its local concentration at the electrochemical surface and ultimately improving the selectivity toward CO_2_ reduction.^73^ After the hydrophobic modification, the faradaic efficiency toward undesired HER decreased from 71% to 10%.^65^ Xing et al. used a very similar strategy consisting of mixing polytetrafluoroethylene with the active material to create a triple-phase boundary of solid–liquid–gas around the active site, suppressing hydrogen evolution.^415^

The dichotomy in hydrophilicity requirements for gas evolution and gas reaction introduces a new challenge in the development of bifunctional OER and ORR catalysts, for integrated fuel cells and electrolyzers. Once again, a possible solution can be found in nature by imitating the breathing process. Alveoli in lungs are covered on the inner side by a layer of lecithin-type hydrophobic molecules to reduce surface tension at the gas interface, while the outer side is hydrophilic to maintain contact with the bloodstream (Figure 16o and p). This asymmetric hydrophobic/hydrophilic membrane ensures efficient gas transport in both directions.^416^ Inspired by this mechanism, Li et al. produced an alveolar structure with a hydrophilic and a hydrophobic side by rolling and sealing a catalyst-coated polyethylene membrane (Figure 16 q and r). This alveolus structure was found to improve both ORR and OER performance compared to that of the same flat electrode.^75^

In conclusion, nature has successfully inspired the development of electrodes with rational and optimized transport of reactants and products, improving the catalytic activity of various electrochemical reactions. However, bioinspiration has guided not only the engineering of active sites and electrodes but also the design of other critical components of electrochemical cells, such as membranes and flow-field plates, which will be discussed below.

## 6 Bioinspired Devices

Bioinspiration does not stop at the catalyst layer but can be advantageous for several parts of an electrochemical device. In this section, we will focus on two fundamental aspects of most electrochemical devices, the flow field and the membrane. The type of membrane depends on the electrochemical device and on the pH at which it operates, but this review will solely focus on proton-exchange membranes, which are the most developed and common to PEM fuel cells, PEM electrolyzers, and CO_2_ reduction electrochemical cells. Section 6.1 will present an overview of both bioinspired and bioderived proton-exchange membranes. Section 6.2 will focus on the flow field, which is common to all electrochemical devices involving gaseous reactants and is responsible for the uniform distribution of gas on the catalyst surface. For its design, researchers have taken inspiration from complex 3D structures that require a similar homogeneous distribution of materials, such as that of sap in leaves or blood in human bodies. Finally, we will discuss flexible electrochemical devices as an example of how bioinspiration can also play a role in the design of electrochemical devices at a macroscale.

### 6.1 Proton-Exchange Membranes

Another critical component of electrochemical devices is the membrane. Depending on the pH, the membrane has the role of transporting hydroxide ions (AEM) or protons (PEM). Recently, research efforts have been focusing on AEMs thanks to the activity of noble-metal-free catalysts for ORR in alkaline conditions. Nevertheless, the ion conductivity and durability of these membranes are still much lower compared to those of PEMs, limiting their widespread adoption. In this section, we will be focusing on proton-exchange membranes, which are common to PEM fuel cells, CO_2_ reduction, and N_2_ reduction cells.

**Figure 17:**
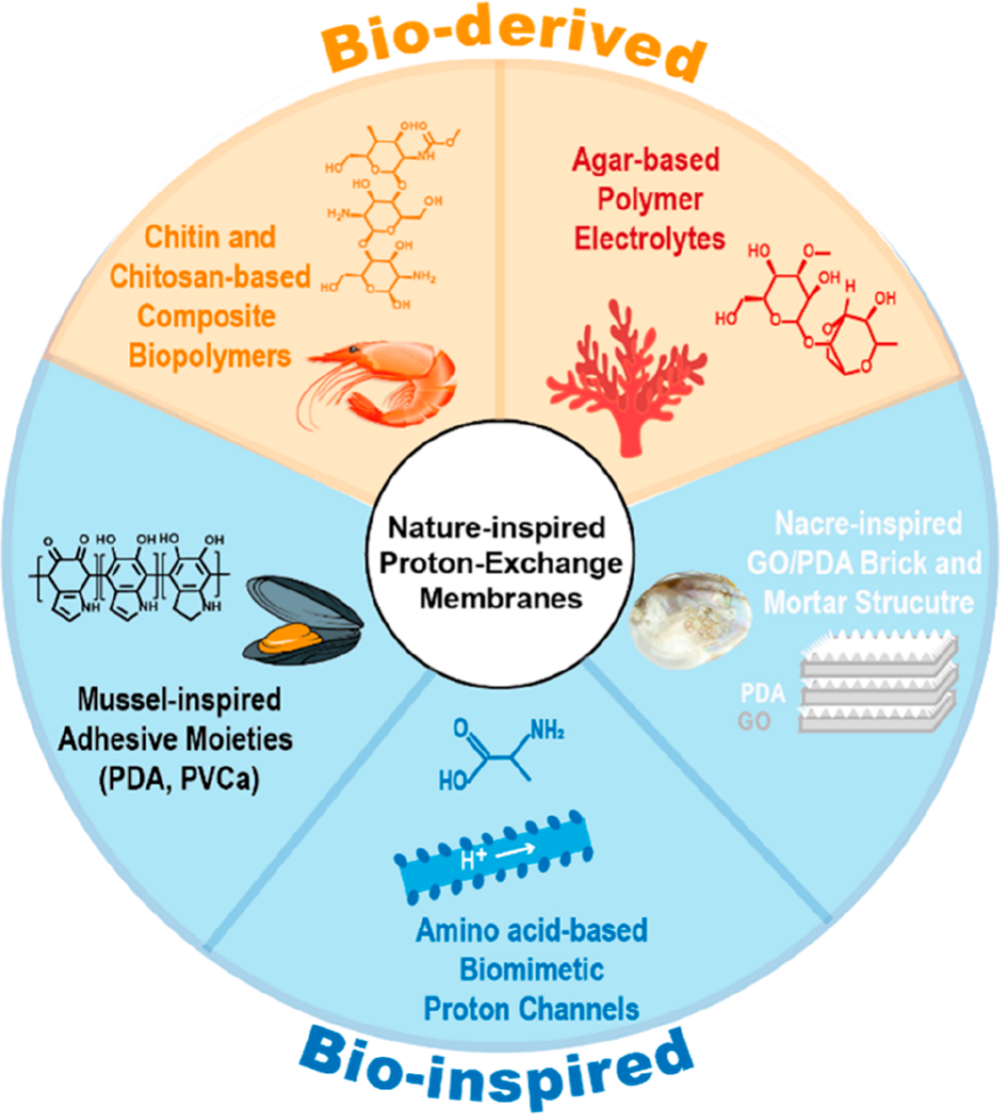
Selection of bioinspired and bioderived materials for PEMs.

Perfluorosulfonic acid (PFSA) membranes are the most used in low-temperature PEMFC, and the most commercially available options are Nafion (Dupont) and Gore-Select (W. L. Gore & Associates). Nafion owes its popularity to its exceptionally high ionic conductivity (6.210^–2^ S cm^–1^ at 100% relative humidity) and durability.^417^ However, PFSA membranes with this membrane have several drawbacks, including high costs, negative environmental impact, toxicity, and low performance at high temperatures or low humidity.^418^ These limitations have driven increased interest in the development of suitable alternatives and, once again, nature has guided this research, both as an inspiration and as a starting material (Figure 17).

One of the most studied natural materials for proton-exchange membranes is chitin, a natural polymer abundantly present in shrimp shells, which are often discarded as waste. Furthermore, chitin is biodegradable, nontoxic, and low-cost.^419^ Yamada and co-workers have used two derivatives of this polymer, chitin phosphate and chitosan, to produce acid–base composite biopolymers as anhydrous proton-conducting membranes. Despite showing lower conductivity compared to Nafion, the so-obtained membranes retained a reasonable conductivity of up to 10^–2^ S cm^–1^ even in absence of water and at temperatures up to 180 °C.^420^ Lupatini et al. reported a conductivity of 1.9 × 10^–2^ S cm^–1^ at 100% relative humidity and 80 °C by cross-linking chitosan with sulfuric acid.^421^ Several other chitosan composites have been reported as polymer electrolytes, such as chitosan and polysulfone,^422^ sulfonated polysulfone,^422^ phosphotungstic acid,^423,424^ phosphomolybdic acid,^425^ zeolites,^426^ sulfonated graphene oxide,^427^ and silicotungstic acid.^428,429^ Finally, Alves and co-workers linked the individual properties of chitosan, such as deacetylation and molar mass, to the proton conductivity of the resulting membrane. They concluded that lower deacetylation and higher molar mass were favorable, with different thicknesses resulting in an order of magnitude increase in proton conductivity.^430^

Another common bioprecursor for the development of membranes is agar-agar, a biodegradable, cheap, and nontoxic polysaccharide derived from red seaweed. Agar-based polymer electrolytes have been developed for applications in several electrochemical devices,^431,432^ including supercapacitors,^433^ dye-sensitized solar cells,^434^ and fuel cells.^435−437^ However, the reported ionic conductivity is around an order of magnitude lower than that of Nafion. Bioinspiration has also led the way to the development of new membranes. The most reported nature-mimicking membranes utilize mussel-inspired adhesive moieties, such as polydopamine and poly(vinyl) catechol. Nagao and co-workers reported the synthesis of nanocomposite films of poly(vinyl) catechol and polystyrene block copolymers, obtaining well-aligned lamellae structures.^438^

Mussel-inspired polydopamine has also been investigated as a universal interfacial cross-linking agent, as it contains bearing abundant −NH_2_ and – NH– groups that can lead to high proton conductivity. This concept has been applied to the synthesis of PEMs with graphene oxide,^439^ SiO_2_,^440^ and CeO_2_.^441^ By modifying the metal–organic framework DHZIF-8 with polydopamine, Rao et al. very recently reported a proton conductivity under anhydrous conditions (120 °C) of 3.66 mS cm^–1^, which is 2.2× higher than that of Nafion.^442^ Taking further inspiration from nature, Cai et al. synthesized a layered proton-exchange membrane based on polydopamine, graphene oxide, and sulfonated poly(vinyl alcohol).^443^ This assembly mimics the brick-and-mortar structure of nacre, with graphene oxide as the brick and the polymers as the mortar. The optimized structure is endowed with a high tensile strength (216.5 MPa, 2.7× higher than that of natural nacre) and excellent proton conductivity (0.303 S.cm^–1^ at 80 °C) and offers higher fuel cell power output and a lower weight compared to Nafion.^443^

Finally, protein-based biomimetic channels were used as inspiration for highly efficient proton transfer.^444^ Most recently, Li and co-workers obtained bioproton channels by incorporating metal–organic frameworks with attached amino acids into a sulfonated polysulfonic matrix. Among the amino acids tested, the glutamate-functionalized MOF demonstrated the highest proton conductivity of 0.212 S cm^–1^ at 80 °C.^445^ Amino acids have also been tested in proton-exchange membranes in conjunction with cellulose whiskers,^446^ chitosan nanofibers,^447^ and cellulose nanofibers.^448^ Finally, protein-based biomimetic channels were used as inspiration for highly efficient proton transfer.^444^

PFSA membranes, in the form of Nafion first and Gore-Select more recently, have been the gold-standard since the 1960s. However, their high cost, toxicity, and limited performance at low humidity have driven research into alternative PEMs. Nafion modification has been extensively used to improve the proton conductivity and stability of the PFSA membrane; however, price and toxicity remain the main drawbacks of this approach. In that sense, bioderived membranes are ideal candidates, as they utilize biodegradable, cheap, and often waste products as starting materials. However, membranes obtained from the popularly studied chitosan and agar display modest proton conductivity. On the other had, bioinspiration has been extremely useful for overcoming some of Nafion’s limitations, such as proton conduction in anhydrous conditions, and offers high tensile strength. Even though significant challenges remain and more research is needed in this area, there are very bright prospects for the commercialization of cost-effective nontoxic proton-exchange membranes, and we are convinced that bioinspiration will play a key role in the development of such materials.

### 6.2 Flow Fields

As discussed in section 3, H_2_ and O_2_ need to be distributed sufficiently and uniformly onto the catalysts surface, thus calling for careful design of the flow field. Bioinspiration can greatly benefit the design of the flow field in electrochemical devices by mimicking apparent characteristic of certain biological structures (leaves, blood vessels, etc.).^449,450^ In 2009, Guessous et al. presented two flow channel designs by imitating the structures of both a leaf and a lung for the first time,^451^ and these findings led to more reports by different groups.^452,453^ However, the lack of a theoretical foundation in these systems led to certain concerns about the scalability or the inhibition of fuel cell performance. Nevertheless several mathematical models have emerged that support such these theories, such as Murray’s Law, fractal theory, and bionic similarity theory.^451,454−456^ Before the designed flow fields were manufactured, computational fluid dynamics modeling was usually performed to simulate the performance of the bioinspired bipolar plate. Some common characteristics of the performance can be the distribution of reactants, the current density, water management, pressure drop, and energy dissipation, among others. Currently, the unique structure of leaves still serves as the most common inspiration source for the flow field design because of its superior ability to uniformly distribute nutrients on a 2D surface. For instance, in 2018, Ouellette et al. reported the effect on direct methanol fuel cells of bioinspired interdigitated and noninterdigitated flow fields compared to a standard serpentine flow field, following their earlier work on Murray’s Law-inspired flow field design.^457,458^ A 3D steady-state, isothermal, and single-phase model was developed to help understand the experimental results obtained under different anode and cathode flow rate combinations. The conventional serpentine design and the interdigitated bioinspired design exhibited the best performance for the anode and the cathode, respectively, due to the enhanced under-rib convection of both flow fields. Ouellette et al. also noted the importance of having an interdigitated design to prevent the reactants from traveling directly from the inlet to the outlet.^419^ Other than the common symmetrical design, asymmetric leaf-shaped flow channels have also been studied. Liu et al. investigated the difference of these two using both a numerical simulation and an experimental study, concluding that an asymmetric bionic flow channel works better when placed perpendicularly with a relatively flat pressure variation.^459^

Another powerful inspiration from nature for flow field design is mammal lungs.^451,455,458^ Recently, Coppens et al.^451,455,458^ developed a model based on the fractal geometry of a lung, with the primary role of distributing reactants homogeneously.^82^ To have diffusion-driven flow equal to the convection-driven flow for even reactant distribution, the optimal generations of branching were found to be four. A 3D-printed large-scale prototype (25 cm^2^) of the design revealed outstanding performance compared to the conventional flow field design, with a 30% improvement of the max power density at 75% RH and the lowest voltage decay (5 mV h^–1^). Following this initial report, neutron radiography was employed to visualize the liquid water distribution of the bioinspired design for the first time.^460^ The results addressed the flooding problem in the interdigitated outlet channels, which leads to performance decay at high humidity conditions and calls for water removal strategies.^460^ More examples have been well documented in reviews focusing specifically on bioinspired flow field design and development.^449,450,461,462^

In summary, despite the manufacturing challenges and the cost, device flow field designs inspired by nature show huge potential to become the new standard. These designs benefit from more efficient water management, enhanced reactant distribution, and reduced pressure drop, resulting in better cell performance comparing to the conventional design.

### 6.3 Flexible Electrochemical Devices

The evolving trend toward wearable electronics has increased the demand for flexible energy devices that can offer a reliable power supply upon bending, stretching, and twisting. To date, research has focused on supercapacitors and Li-ion batteries, which will not be covered in this review; readers may refer to relevant review articles.^463−467^ Most recently, flexible fuel cells have received increased attention thanks to their high energy density and fast recharging times compared to Li-ion batteries. Several types of flexible fuel cells have been reported, including biofuel, proton-exchange membrane, and photocatalytic fuel cells.^468^

Once again, bioderived precursors have proven advantageous for the manufacture of such materials. For wearable devices, the flexibility of all components, such as the electrode, the separator, and the current collector, is essential. The electrodes are especially important to the flexibility of the device and complex to produce. Fiber-based materials are among the most promising catalyst supports thanks to their high flexibility and deformability. Paper has been widely investigated as an electrode support,^469,470^ particularly for microfluidic fuel cells, as the natural capillary structure of paper can drive the flow, removing the need for an external pump.^471−474^ For example, Chan and co-workers fabricated a flexible, membrane-less hydrogen peroxide microfuel cell on paper. Exploiting paper’s unique features such as flexibility, porosity, and capillarity, they obtained a power density of 0.81 mW cm^–2^ at 0.26 V even after the distortion of the cell. Additionally, cellulosic paper is abundant, low-cost, and biocompatible, making it an ideal candidate for flexible and sustainable fuel cells.^472^ Similarly, Wang et al. reported 75–87% performance retention upon bending a paper-based hydrogen fuel cell from 45° to 135°, reaching a peak power density of 4 mW cm^–2^.^470^ Paper-based fuel cells have also been reported using formate as a fuel, achieving a maximum power density of 2.5 mW mg_Pd_^–1^.^474^ Besides paper, Zhang et al. reported the use of cotton in the production of a flexible nanocomposite membrane for application in microbial fuel cells. Once again, the use of a natural fiber, such as cotton, offers low costs and low toxicity but also allows high proton conductivity and a higher current density, compared to Nafion, in microbial fuel cells, demonstrating a peak power of 400 mWm^–2^.^475^

Silk fibroin, the main protein constituent of silk fibers of *Bombyx mori* silkworms, is another example of bioderived materials for flexible fuel cells. In a recent report, Tseng et al. successfully utilized silk fibroin–carbon nanotube composites in the production of biocatalytic fuel cells.^476^ In this assembly, silk fibroins act as binders by mechanically trapping enzymes and improving the electrical connection between the enzymes’ active sites and the carbon nanotubes, enabling the formation of flexible electrodes with enhanced durability. Even though they have not yet been applied to fuel cells, transparent and flexible microstructured surfaces have also been synthesized from chitin. As mentioned before in this review, chitin is a natural, abundant, and biodegradable amino-polysaccharide that can be extracted from the exoskeletons of shrimps and crabs, which are often discarded as waste.

Despite recent interest and improvement, at present, flexible fuel cells suffer from low durability, particularly upon bending and twisting, which can decrease the adhesion of the catalyst to the electrode and change its mechanical structure. We believe that nature can help the design of bendable fuel cells the same way it helped the design of flexible energy storage and conversion devices. For example, flexible Li-ion batteries have been synthesized by mimicking the structure of an animal spine, with alternating thick stacks of the electrode and an unwound part corresponding to vertebrae and soft marrow, respectively.^477^ Taking inspiration from snake skin, where rigid keratin scales are interconnected with flexible hinges, shape-forming batteries consisting of hexagonal, rigid unit cells with flexible electrical interconnections have also been reported .^478^ Flexible phototermo-supercapacitors have also benefited from looking at natural structures, for example, that of nacre, where mechanical robustness is achieved by a mortar-and-brick structure.^479^

Other examples include leaf-skeleton-inspired supercapacitors and HER photocatalysts,^480,481^ vertebral- and nacre-inspired brick-and-mortar design for solar cells,^482,483^ and biomimetic interlocking structures for interfacially strengthened flexible supercapacitors.^484^ Flexible pressure sensors with improved sensitivity have also been reported using a range of biotemplates, such as aureum leaves,^485^ rose petals,^486^ banana,^487^ mimosa,^488^ and lotus leaves.^489^ These examples demonstrate how nature can offer valuable insights and pave the way for the development of flexible energy devices.

## 7 Conclusions and Outlook

In this review, we summarized how nature can inspire solutions to energy conversion technologies by providing natural feedstock and inspiration. Biomass can be employed for the preparation of advanced carbonaceous materials, and enzymes serve as models of highly efficient electrochemical systems in scenarios ranging from water splitting or oxygen reduction to CO_2_ conversion. Despite the recent advancement in the field in terms of bioinspired catalyst preparation or the implementation of bioinspired devices, there is still a long way to go before nature can make a tangible impact in modern energy conversion devices. Based on the current state-of-the-art, and with the aim of advancing toward nature-inspired and derived devices, we believe that upcoming research should tackle the following challenges:

1. Rigorous electrochemical testing protocols. When testing any electrocatalyst, one should make sure standardized tests take place and that the reported data are compared to a rigorously benchmarked common reference catalyst (Pt/C in ORR, IrOx in OER, etc.) with sufficient provided experimental details. We would like to refer the reader to previously published reviews that address best practices and the growing concern for the proper assessment of electrocatalytic measurements.^490−493^
2. Reliable synthetic protocols for the preparation of bioinspired catalysts from biomass. Biomass-derived carbon materials display, in general, high overpotentials for water splitting half-reactions, CO_2_ reduction, and 4e^–^ ORR owing to their metal-free character. Additionally, biomass-derived catalysts can be obtained in many different complex nanostructures, making it difficult to screen for standardized structure–activity relationships, as recently highlighted.^494^ Through the rational selection of heteroatom-containing biomass precursors and reaction conditions, such as using active site templates,^21,243,244^ a biomass-derived carbon-based material could be engineered in terms of chemical composition and porosity, leading to a suitable substrate for the coordination of atomic metallic species to form electrochemically active sites such as single-atom M–N_*x*_, which resembles heme. Meanwhile, the next generation of nature-inspired heterogeneous electrocatalysts should incorporate 3D dual-metal-atom active sites resembling enzymes, such as CcO for ORR, which can achieve high TOFs with lower reaction overpotentials than Pt and PGM alloys. Directly employing enzymes in practical devices is not feasible due to their orders of magnitude lower site densities and limited stability in harsh but practical device environments. Emulating their 3D active-site structure would require a well-controlled synthetic pathway, which is likely not possible with biomass. Instead, new classes of materials are required that can withstand practical device conditions with sufficient porosity and conductivity while maintaining a 3D structure; this can possibly be achieved using ordered carbonaceous frameworks^288,289^ or carbon materials derived from tailored metal organic frameworks.^286,495^
3. Assessment of the bioinspired active site structure and degradation. The structure of bioinspired active sites that contain single- or dual-metal atoms is highly challenging and often leads to controversy in the field. For advanced characterization techniques suitable for differentiating between these kind of active sites, we would like to refer the reader to our previously published review.^184^ Recent advances include work by Mitchell et al., who reported a deep-learning method for the automated detection of single atoms in a TEM that overcomes the poor statistical significance and reproducibility inherent of manual operation.^496^ Meanwhile, the stability and degradation of atomic active sites remain relatively unexplored to date; therefore, emphasis should be placed on developing in situ techniques. The importance of applying known techniques in novel ways was recently highlighted by Elbaz and co-workers, who for the first-time applied Fourier-transform alternatin- current voltammetry to probe the active site density and degradation of a Fe-NC ORR catalyst in situ in a PEMFC.^497^ Additionally, thorough post-mortem characterization (for example, XPS, identical location microscopy, XAS, etc.) of the catalyst should be provided to confirm active-site stability.
4. Self-healing. In OER, the catalytic self-repairing PSII is firmly established via cluster reassembly of the core active sites. While this complex structural process has not been successfully mimicked to date, continually regenerating metal oxide OER systems have been established, where activities of the species are restored through dynamic equilibrium dissolution–redeposition cycles under specific operational conditions.^498^ By increasing the understanding of this process, an ideal self-healing catalyst could be achieved via instantaneous redeposition coupled with structural stabilization. Meanwhile, for ORR, nature’s ability to regenerate has some similarities to the ability of single-atom M–NC catalysts to become reactivated following thermal pyrolysis or electrochemical reduction.^316,317^ Further understanding the mechanism of this process could help extend of the life of these catalysts and move from current lifetimes (∼100 h) toward practical device lifetimes (>5000 h).
5. Improvement of electron, mass, and proton transport in electrochemical devices. In contrast to enzymes, where every molecule involved in the reaction has a separate and well-defined pathway, in most electrochemical devices the products and reactants diffuse through the same electrolyte in opposite directions. This lack of order for mass, electron, and proton transport limits the overall performance of these devices and should be tackled starting from nature. One way to approach this problem is to tailor the hydrophobicity of the catalytic surface. This has been successfully achieved for both gas-evolving and gas-consuming reactions, inspired by, among others, the hydrophilic scales of fishes^399^ and the hydrophobic skin of diving spiders.^73^ Proton transport can also be rationally improved by mimicking the outer coordination spheres of enzymes.^76^ We believe that the imitation of proteins channels in enzymes still holds a great potential for the rational control of product and reactant transport to the active site.
6. Development of low-cost, nontoxic, and high-performing proton-exchange membranes. The benchmark perfluorosulfonic acid proton-exchange membranes offer great proton conductivity but are expensive, toxic, and low-performing at low humidity. Regarding the first two limitations, bioderived membranes are excellent replacements, as they can offer cheap and biodegradable starting materials. On the other hand, bioinspiration can improve proton conductivity in a wider range of temperatures and humidity, as shown by the recent development of nature-inspired anhydrous proton-conducting membranes.^420^
7. Evaluation of the sustainability and scalability of bioderived catalysts. Despite the vast amount of literature on developing bioderived and bioinspired catalysts, currently very few systematic evaluation methods have been developed to assess the impact of transforming biomass into catalysts across all life stages. In addition, although biomass is a renewable resource, the impact of excessive sourcing presents a point of important consideration in regard to ecosystem damage. This will in turn affect the scale-up of systems for catalyst manufacturing. Therefore, sustainable, and socioeconomic frameworks need to be established to benefit the development of relevant technoeconomic analysis and life cycle assessment models. Based on such frameworks, robust analysis needs to be performed and benchmarked to the utilization of conventional raw materials, considering biomass sourcing, extraction, pretreatment, and synthesis steps.
8. Rational design and applicability of the hierarchical catalyst structure. Despite the vast research carried out on developing catalyst architectures via either directly employing the hierarchical biomaterials or fabricating structures inspired by the nature, remarkable challenges remain. First, there is a lack of rationale behind the designs of the structures. The ideal porosity or architecture required by the reaction systems is still undetermined and unable to guide the construction of catalysts. Computational modeling should be combined with careful material synthesis to clarify the requirements. Moreover, current catalyst development often results in difficult application in real devices due to various reason,s including tedious synthesis processes, fragile products, and impracticable scale-up procedures. All these factors need to be tackled thoroughly to fully utilize bioinspired hierarchical catalysts.

## List of Abbreviations

ORR: oxygen reduction reaction
HER: hydrogen evolution reaction
GHG: greenhouse gases
PEM: proton-exchange membrane
OER: oxygen evolution reaction
PCET: proton-coupled electron transfer
OEC: oxygen-evolving complex
TOF: turnover frequency
MWCNT: multiwalled carbon nanotubes
GDE: gas diffusion electrode
MEA: membrane–electrode assembly
DFT: density functional theory
EXAFS: extended X-ray absorption fine-edge structure
XANES: X-ray absorption near-edge structure
SD: site density
PGM: platinum-group metal
CO2RR: CO_2_ reduction reaction
CODH: CO dehydrogenase
SEM: scanning electron microscopy
AEM: anion exchange membrane
MOF: metal–organic framework
PFSA: perfluorosulfonic acid
RH: relative humidity
